# Replication timing alterations are associated with mutation acquisition during breast and lung cancer evolution

**DOI:** 10.1038/s41467-024-50107-4

**Published:** 2024-07-18

**Authors:** Michelle Dietzen, Haoran Zhai, Olivia Lucas, Oriol Pich, Christopher Barrington, Wei-Ting Lu, Sophia Ward, Yanping Guo, Robert E. Hynds, Simone Zaccaria, Charles Swanton, Nicholas McGranahan, Nnennaya Kanu

**Affiliations:** 1 Cancer Research UK Lung Cancer Centre of Excellence, University College London Cancer Institute, London, UK; 2 Cancer Genome Evolution Research Group, Cancer Research UK Lung Cancer Centre of Excellence, University College London Cancer Institute, London, UK; 3 Cancer Evolution and Genome Instability Laboratory, The Francis Crick Institute, London, UK; 4 Computational Cancer Genomics Research Group, University College London Cancer Institute, London, UK; 5 Department of Oncology, University College London Hospitals, London, UK; 6 Bioinformatics and Biostatistics Science Technology Platform, The Francis Crick Institute, London, UK; 7 Advanced Sequencing Facility, The Francis Crick Institute, London, UK; 8 CRUK Flow Cytometry Translational Technology Platform, UCL Cancer Institute, London, UK

**Keywords:** Cancer genomics, Breast cancer, Non-small-cell lung cancer, Evolutionary genetics

## Abstract

During each cell cycle, the process of DNA replication timing is tightly regulated to ensure the accurate duplication of the genome. The extent and significance of alterations in this process during malignant transformation have not been extensively explored. Here, we assess the impact of altered replication timing (ART) on cancer evolution by analysing replication-timing sequencing of cancer and normal cell lines and 952 whole-genome sequenced lung and breast tumours. We find that 6%–18% of the cancer genome exhibits ART, with regions with a change from early to late replication displaying an increased mutation rate and distinct mutational signatures. Whereas regions changing from late to early replication contain genes with increased expression and present a preponderance of APOBEC3-mediated mutation clusters and associated driver mutations. We demonstrate that ART occurs relatively early during cancer evolution and that ART may have a stronger correlation with mutation acquisition than alterations in chromatin structure.

## Abstract (web summary)

The role of altered replication timing (ART) during malignant transformation requires further exploration. Here, analysis of replication-timing sequencing and whole genome sequencing reveals a significant association of ART with the genomic and transcriptomic landscape during cancer evolution in lung and breast tumours.

## Introduction

Cancer development is an evolutionary process where mutations serve as a substrate upon which selection can act. Thus, understanding the mechanisms underlying mutational accumulation is crucial to illuminate the processes that shape tumour evolution^1^. DNA replication during each cell cycle is an essential biological process that involves the duplication of the entire genome faithfully^2^. To ensure efficient and accurate replication, and to limit the potential for acquisition of somatic alterations, each chromosome is divided into segments that are replicated in a defined and highly organised temporal order, termed the replication timing (RT) programme^2,3^. In non-malignant cells, the RT programme is highly conserved across 50%–70% of the genome, while the remaining 30%–50% can dynamically vary during normal development, contributing to tissue-specificity^3^. Changes to the RT programme during normal lineage differentiation are associated with differences in the transcription level of genes^4^.

The RT programme has been linked to the non-uniform acquisition of somatic mutations across the genome during cancer development^5^. Multiple studies have shown that late replicated regions, which often coincide with condensed chromatin regions, accumulate more mutations including both single nucleotide variants (SNVs)^6–8^ and somatic copy number alterations (SCNAs)^9–11^, compared to regions replicated early during S phase which often are actively transcribed and exhibit open chromatin^6,12^. Linked to this, prior work has revealed that the patterns of mutational signatures observed across the genome are strongly associated with the RT programme^13^.

However, despite the association between the RT programme and the overall genetic alterations in cancer, few studies have focused on how the plasticity and changes to the RT programme interact with the genomic landscape in cancer and thus contribute to cancer development^4,14–16^. Changes to the RT programme between acute lymphoblastic leukaemia cells were reported to be much smaller than those between leukaemia cells and matched normal lymphocytes, suggesting dramatic alterations to RT in cancer compared to normal cells^14,15^. Similar results were reported in prostate adenocarcinoma compared to matched prostate epithelial cells, with RT changes coinciding with specific types of translocations in cancer^16^.

The potential mechanisms responsible for the association between late RT and increased genetic alterations remain unclear. Given the link between chromatin configuration and RT^3,17–19^, the elevated mutation rate in late replicated regions could primarily reflect a condensed chromatin structure and low DNA repair activity^20^. However, an in-depth evaluation of the relative impact of changes in chromatin structure versus alterations in RT during cancer development is lacking. Further, the extent to which alterations to the RT programme are associated with the activity of mutational processes remains unclear.

Here, we set out to explore the extent to which replication timing changes during malignant transformation and its correlation with mutation acquisition, gene expression and chromatin structure during cancer evolution.

## Results

### A resource to evaluate replication timing in lung and breast cancer

To evaluate the dynamics of mutation acquisition during cancer evolution, we first explored the density of mutations across the genome by analysing 952 cancer genomes derived from the 100,000 Genomes Project^21^ and Nik-Zainal et al.^22^. This cohort consists of 470 lung adenocarcinoma (LUAD) and 482 breast carcinoma (BRCA) tumours, containing 152 triple negative breast carcinomas (TNBC), 266 HER2-negative (HER2-), 22 HER2-positive (HER2+) and 42 other breast carcinomas (“Methods” section).

**Fig. 1:**
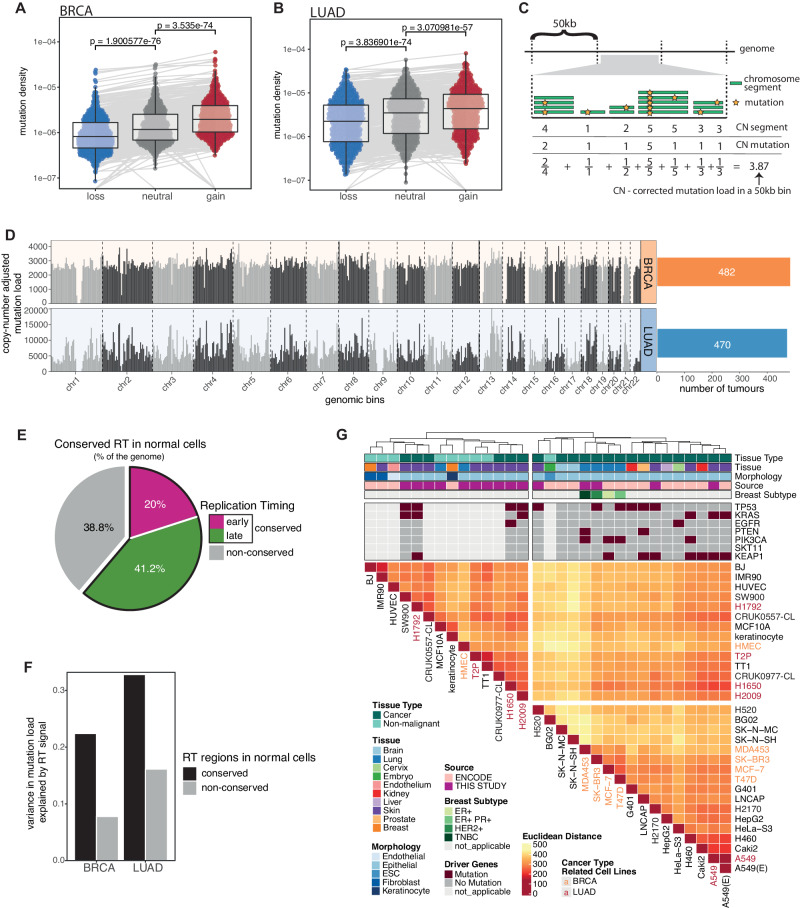
Overview of the data cohort used to explore the relationship between mutation acquisition and replication timing. **A**, **B** Mutation density (measured as the number of mutations relative to the size of the affected genomic regions) in gained, lost and copy number neutral genomic regions in 482 breast carcinomas (BRCA) (**A**) and 470 lung adenocarcinomas (LUAD) (**B**). *P*-values reflect one-sided paired Wilcoxon tests. The centre line of the box plots represents the median value, the limits represent the 25th and 75th percentile, and the whiskers extend from the box to the largest and lowest value no further than 1.5 * IQR (interquartile range) away from the box. **C** Schematic demonstrating the method of copy number correcting the mutation load within a single 50 kb bin. Both the total copy number at the mutated position (referred to as CN segment) and the number of mutated alleles (referred to as CN mutation) are calculated. **D** Copy number adjusted mutation load in 5 Mb bins across the genome for 482 BRCA and 470 LUAD tumours. **E** Fraction of the genome presenting conserved and non-conserved RT across 5 non-malignant cell lines from ENCODE. **F** Variance in mutation load explained by the average replication timing (RT) signal across all 16 ENCODE cell lines in conserved and non-conserved RT regions identified across non-malignant cells. The bars represent the *R*^2^ value derived from a linear model with mutation load as an independent variable and the averaged RT signal as a dependent variable. **G** Hierarchical clustering of RT signals in 50 kb windows across the genome. The Euclidean distance and the ward criterion were used to cluster RT signals of 31 cell lines (including 15 IN-STUDY and 16 ENCODE cell lines). Additional information about the cell lines, including whether the cell line was derived from normal or cancer cells and the presence of different driver gene mutations, are displayed on the top tracks. Names of a subset of cell lines were coloured regarding their involvement in the corresponding cancer type in further analyses. A549 was Repli-sequenced as part of both the ENCODE and our IN-STUDY cohort representing two replicates; A549(E) presents the results using the ENCODE data.

Comparing the mutation density in genomic regions harbouring different types of somatic copy number alterations (SCNAs) revealed a significantly elevated mutation rate in genomic regions subject to copy number (CN) gains relative to ploidy compared to those without (Fig. 1A, B). This suggests that previous studies examining the density of mutations across the genome may be confounded by ignoring the impact of SCNAs on mutation acquisition. To exclude this potential bias, we, therefore, adjusted the local mutation burden (*i.e*., the mutational burden in a given region of the genome) by the estimated number of alleles harbouring each mutation relative to the total copy number at that position (Fig. 1C). The mutational density across the genome was then calculated as the adjusted number of mutations in 50 kb windows for the aggregated set of mutations for all BRCA and LUAD tumours respectively (Fig. 1C; “Methods” section). Nevertheless, even after correcting for underlying copy number alterations, we observed a high variability in local mutation burden across the whole genome (BRCA: mean = 2717.88, standard deviation (SD) = 539.51; LUAD: mean = 6511.58, SD = 3579.71) and between cancer types (Fig. 1D).

To explore the extent to which this variability in local mutation burden could be explained by replication timing, we obtained replication-timing sequencing (Repli-seq) data from different tissue types and cell differentiation states from ENCODE (“Methods” section)^23,24^. In total, we obtained Repli-seq data from 16 cell lines including 5 non-malignant (“normal”) and 11 malignant cell lines. Broadly, the genome could be divided into regions exhibiting conserved RT (where all five non-malignant cell lines exhibited concordant early or late replication timing) or non-conserved RT (where we observed discordance in replication timing between the cell lines) (Fig. 1E). In both BRCA and LUAD, we observed that RT in conserved regions was better able to predict local mutation burden than RT in non-conserved regions (Fig. 1F). Therefore, we reasoned that changes to the RT programme that occur during the malignant transformation from normal to tumour cells may also correlate with mutation acquisition during tumour development.

Given the tissue-specificity of the RT programme^16,25^, an evaluation of RT changes during malignant transformation requires RT information from malignant and their matched non-malignant cells derived from the same tissue-of-origin. However, most publicly available RT cancer datasets do not include paired non-malignant samples derived from the tissue-of-origin of the cancer sample. Therefore, to systematically investigate the association of RT and RT changes in cancer, we conducted Repli-seq experiments for 2 BRCA, 4 LUAD, 3 LUSC, 2 patient-derived cell lines (PDCs) from two TRACERx LUAD tumours, as well as 4 immortalised non-transformed (non-malignant as “normal”) cell lines including cells that were derived from the likely originating tissue (referred to as tissue-of-origin) of BRCA (*i.e*., human mammary epithelial cells (HMEC)) and LUAD (*i.e*., pulmonary alveolar epithelial type II cells (T2P)) (“Methods” section; Supplementary Fig. 1 and Supplementary Table 1). High concordance, robustness, and reproducibility within and between the data generated as part of this study (IN-STUDY) and ENCODE data were observed when comparing the RT signal of a subset of biological replicates in this comprehensive RT cohort (Supplementary Fig. 2; “Methods” section).

To evaluate the extent to which the RT programme is different in cancer cells from their normal counterparts, we performed unsupervised hierarchical clustering of the RT signals of 9 non-malignant and 22 cancer cell lines from different organ sites (ENCODE and IN-STUDY data) and calculated the clusterwise Jaccard bootstrap mean to measure the stability of our clustering results (Fig. 1G). The overall fraction of early versus late replicated genomic regions was similar across all normal and cancer cells (mean 38% early and 62% late) (Supplementary Fig. 3A). However, consistent with previous work^16^, we identified two distinct RT groups, separating normal from cancer cells (Jaccard bootstrap mean: 0.77 for the normal cluster and 0.78 for the cancer cluster), suggesting fundamental changes to RT profiles during malignant transformation from normal to cancer cells.

### Replication timing alterations in cancer are recurrent and correlate with mutation acquisition

**Fig. 2:**
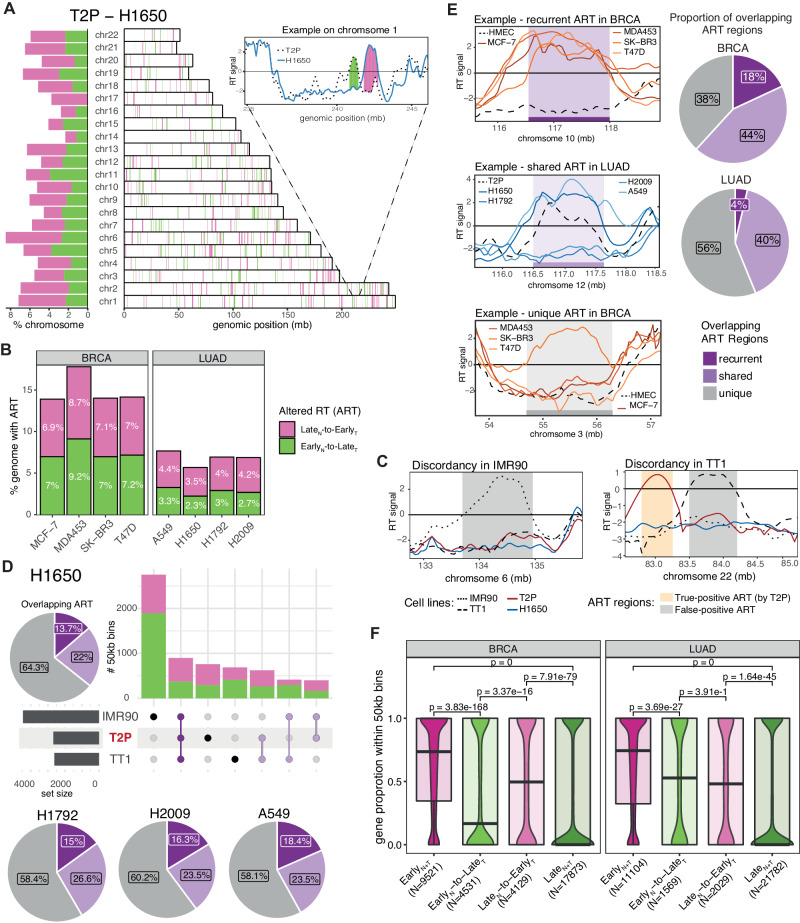
Replication timing alterations in BRCA and LUAD cell lines. **A** Distribution of ART across the genome for one lung adenocarcinoma (LUAD) cell line, H1650. The bars on the left illustrate the proportion of each chromosome affected by altered replication timing (ART). The bars on the right present the localisation of genomic regions with ART on each chromosome. One genomic region on chromosome 1 is displayed to highlight the definition of altered Early_N_-to-Late_T_ and Late_N_-to-Early_T_ replicated regions. **B** Proportions of the genome affected by ART in each of the breast carcinoma (BRCA) and LUAD cell lines. **C** Two examples illustrating how IMR90 and TT1 result in distinct ART regions when used as normal reference for H1650 cells. The regions presented as grey rectangles can be considered false-positive ART regions. In contrast, the yellow rectangle shows a true ART region which has been missed by IMR90 and TT1 (false negative). **D** Proportion of overlapping ART regions that have been identified when using three different cell lines as a reference (IMR90, TT1 and cells most closely resembling the reported tissue-of-origin for LUAD, T2P). The proportions are displayed as pie charts for the 4 different LUAD cell lines with the upset plot present for the H1650 cell line. **E** Pie charts showing the proportions of overlapping ART regions between cell lines within each cancer type by using the correct tissue-of-origin as normal reference, and line charts showing examples of genomic regions with recurrent, shared and unique ART. **F** Comparisons of gene density between genomic regions with unaltered replication timing or shared and recurrent ART in BRCA and LUAD cell lines (BRCA: 9521 genes with unaltered Early_N+T_ replication timing, 4531 genes with Early_N_-to-Late_T_ ART, 4129 genes with Late_N_-to-Early_T_ ART and 17873 genes with unaltered Late_N+T_ replication timing; LUAD: 11104 genes with unaltered Early_N+T_ replication timing, 1569 genes with Early_N_-to-Late_T_ ART, 2029 genes with Late_N_-to-Early_T_ ART and 21782 genes with unaltered Late_N+T_ replication timing). The centre line of the box plot represents the median value, the limits represent the 25th and 75th percentile, and the whiskers extend from the box to the largest and lowest value no further than 1.5 * IQR (interquartile range) away from the box. *P*-values reflect two-sided paired Wilcoxon tests.

Next, to evaluate specific genomic regions exhibiting alterations to the RT programme in BRCA and LUAD, we compared the RT signal of each cancer cell line to a non-malignant cell line derived from the likely matched tissue-of-origin for both cancer types^26,27^. Regions of the genome with altered replication timing (ART) were classified as either Late_N_-to-Early_T_ (late replicated in normal, but early replicated in cancer) or Early_N_-to-Late_T_ (early replicated in normal, but late replicated in cancer) replicated, while other genomic regions with no change in replication timing were classified as Early_N+T_ or Late_N+T_ replicated (Fig. 2A; Supplementary Fig. 3B–D; “Methods” section).

On average, 5.7% of the cancer genome was subject to Late_N_-to-Early_T_ shifts (range, 3.5%–8.7%) and 5.2% to Early_N_-to-Late_T_ shifts (range, 2.3%–9.2%) across LUAD and BRCA cell lines (Fig. 2B). These proportions are consistent with previous findings in other cancer types^14,16^. Most conserved RT regions identified in the four non-malignant cell lines in our IN-STUDY cohort were also conserved among all five non-malignant cell lines from ENCODE (Supplementary Fig. 4A). Furthermore, the majority of ART regions in BRCA and LUAD coincided with non-conserved RT regions across non-malignant cells (Supplementary Fig. 4B, C).

Notably, only an average of 15.9% of ART regions (range 13.7%–18.4%) were concordantly classified as subject to ART across all LUAD cell lines when using three different non-malignant lung cell lines which were derived from the matched (*i.e*., T2P) or unmatched (*i.e*., TT1 and IMR90) originating cell type as a reference (Fig. 2C, D, Supplementary Fig. 5A–D). Similar results were observed in BRCA when comparing ART regions identified with the matched (HMEC) versus unmatched (MCF10A) non-malignant cells (mean 22.0%, range 22.6%–35.2%) (Supplementary Fig. 5E–H). These results highlight the importance of using an appropriate control to identify ART regions in cancer cells.

To identify genomic regions that are recurrently prone to ART across samples within the same cancer type, we evaluated the cancer type specific overlap of ART regions across cancer cells (Supplementary Fig. 5I, J). The observed numbers of overlapping ART regions in 50 kb bins were significantly higher than expected within both LUAD and BRCA cells (Supplementary Fig. 5K; “Methods” section). Intriguingly, although the four different BRCA cell lines were derived from different breast cancer subtypes, their RT profiles were highly correlated (Fig. 1G; Pearson correlation coefficient range 0.76–0.86; *p*-values < 2.2e−16), with 18% of ART regions recurrently identified in all four BRCA cell lines (recurrent ART) and 44% identified in at least two but not all cell lines (shared ART) (Fig. 2E; “Methods” section). In comparison, 40% of ART regions were shared and only 4% were recurrently altered among the four LUAD cell lines (Fig. 2E, Supplementary Fig. 5I, J).

In both BRCA and LUAD, we observed that genomic regions subject to Late_N_-to-Early_T_ alterations in cancer exhibited a higher density of genes compared to regions with unaltered Late_N+T_ or Early_N_-to-Late_T_ replication timing (Fig. 2F) which might explain the observed non-uniform distribution of ART regions across the genome (Fig. 2A; Supplementary Fig. 6).

To explore the association between both unaltered RT and ART with the mutation distribution across the genome in cancer, we harnessed the mutation data from the 952 lung and breast cancer genomes. We first compared the copy number corrected mutation load in genomic bins between different unaltered RT and ART regions in BRCA and LUAD. As expected, and consistent with previous work, we observed an increased mutation load in late compared to early replicated regions in both lung and breast cancer (Supplementary Fig. 7A)^28,29^. We also observed a significantly higher mutation load in Early_N_-to-Late_T_ replicated regions compared to unaltered Early_N+T_ regions and a significantly lower mutation burden in Late_N_-to-Early_T_ regions compared to unaltered Late_N+T_ regions (Supplementary Fig. 7A). These results suggest that ART has a significant correlation with mutation accumulation during tumour evolution, with Late_N_-to-Early_T_ regions potentially providing protection from mutagenesis.

**Fig. 3:**
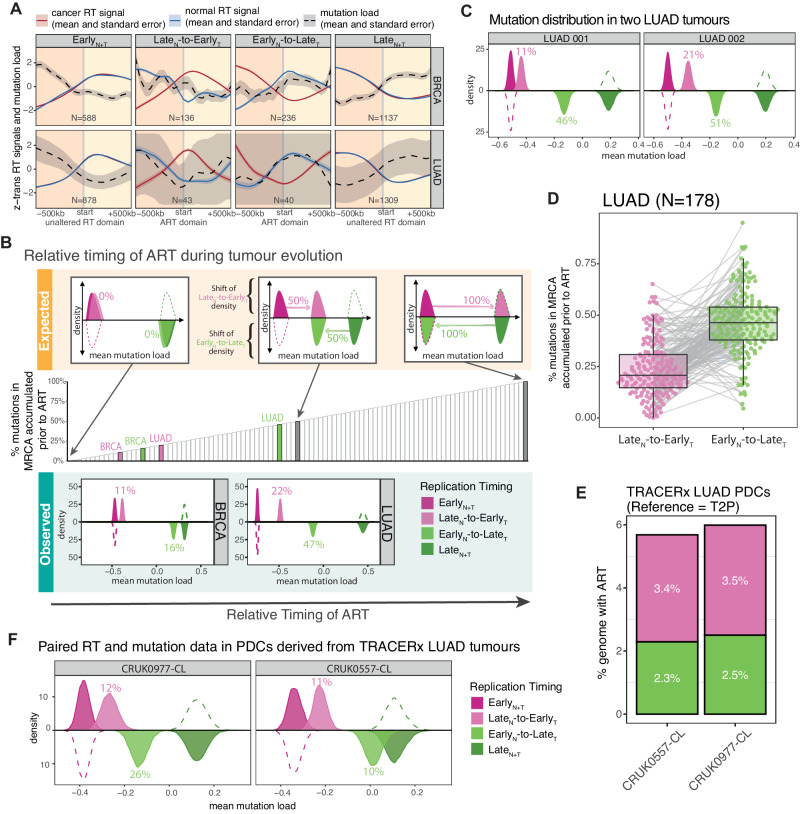
The correlation of ART with the mutation distribution across the genome in BRCA and LUAD tumours. **A** The association between average replication timing (RT) signals and mutation load in genomic regions 500 kb before and after the start of an unaltered RT or recurrently altered replication timing (ART) domain in breast carcinomas (BRCA) and lung adenocarcinomas (LUAD). **B** Expected and observed bootstrapped mean mutation load distributions in ART and unaltered RT regions, indicating the timing of ART occurrence relative to mutation accumulation in the most recent common ancestor (MRCA). Upper plots: Expected patterns illustrating how the relative timing of ART should be captured by the number of mutations occurring before or after ART. The distribution in Early_N_-to-Late_T_ regions is expected to be the same as in Late_N+T_ regions when all mutations in the MRCA are accumulated after ART while the distribution is expected to move closer to the distribution in Early_N+T_ regions when more mutations are accumulated before ART. Lower plots: Observed distributions of mean mutation load values in different altered and unaltered RT regions per cancer type and their estimated ART timing relative to the mutation accumulation in their MRCA. Middle plot: Bars represent percentages of mutations accumulated prior to ART with percentages from the upper plots highlighted in grey. **C** The bootstrapped mean mutation load distributions in ART and unaltered RT regions in two LUAD tumours as examples. **D** Estimated proportions of mutations that were likely accumulated before Early_N_-to-Late_T_ or Late_N_-to-Early_T_ changes in 178 individual LUAD tumours that present a significantly different mutation density in unaltered Early_N+T_ versus Late_N+T_ regions. The centre line of the box plot represents the median value, the limits represent the 25th and 75th percentile, and the whiskers extend from the box to the largest and lowest value no further than 1.5 * IQR (interquartile range) away from the box. **E** Proportions of the genome presenting Early_N_-to-Late_T_ and Late_N_-to-Early_T_ alterations in patient-derived cell cultures (PDCs) from two LUAD tumours from TRACERx patients. **F** Bootstrapped mean mutation load distributions in ART and unaltered RT regions using mutation and RT information from the same cell line.

To explore whether a difference in the mutation distribution could be observed at the boundaries of ART, we analysed the average mutation distribution from 500 kilobase pairs (kb) upstream to 500 kb downstream of the start of unaltered RT and recurrent ART regions. Given that RT is regulated at the replication domain level^30^, we combined multiple adjacent genomic bins displaying the same unaltered RT or ART classification into one RT domain and used the start of this domain for this analysis (“Methods” section). A clear difference in the mutation load was observed at the start of different unaltered RT and ART domains, with an increased mutation load in Early_N_-to-Late_T_ replicated regions but a decreased mutation load in Late_N_-to-Early_T_ regions (Fig. 3A). These data indicate that the mutation distribution in ART regions predominantly reflects the resulting RT in cancer (*i.e*., after the RT change), rather than the RT in the tissue-of-origin (*i.e*., before the RT change).

### RT alterations occur early during cancer development

Conceivably, if ART occurs before any mutations have accumulated (*i.e*., very early during tumour development), Early_N_-to-Late_T_ regions should harbour a similar mutation burden to unaltered Late_N+T_ regions, while the mutation burden in Late_N_-to-Early_T_ regions should be equivalent to unaltered Early_N+T_ (Panel labelled as “expected” in Fig. 3B). Conversely, if ART occurs late during tumour evolution (*i.e*., after the majority of somatic mutations have accumulated), the mutation burden in Late_N_-to-Early_T_ genomic regions should appear equivalent to unaltered Late_N+T_ and the mutation burden in Early_N_-to-Late_N_ should be similar to unaltered Early_N+T_ (Panel labelled as “expected” in Fig. 3B).

To quantify whether ART occurred at different time points during tumour evolution in BRCA and LUAD, we applied a bootstrapping approach to compare the average truncal mutation load (*i.e*., mutations present in the most recent common ancestor (MRCA) of all cancer cells and prior to subclonal diversification) between different unaltered RT and ART regions (Fig. 3B, “Methods” section).

Simulating ART to occur at different epochs of tumour evolution revealed that the observed differences in the mutation frequency between ART and unaltered RT regions can be used to estimate the proportion of mutations that have accumulated before ART during tumour evolution (Supplementary Fig. 8; “Methods” section). Applying this approach revealed that in BRCA, approximately 11% of mutations were accumulated before Late_N_-to-Early_T_ changes and 16% before Early_N_-to-Late_T_ changes. Thus, we speculate that the majority of mutations likely occur following ART changes. Likewise, in LUAD, it was estimated to be 22% for Late_N_-to-Early_T_ changes and 47% for Early_N_-to-Late_T_ alterations (Panel labelled as “observed” in Fig. 3B). These data also suggest that in molecular evolution time, the Late_N_-to-Early_T_ changes potentially occur earlier than the Early_N_-to-Late_T_ changes in both BRCA and LUAD. Repeating the same analysis within each BRCA subtype confirmed that the observed similarities between unaltered and altered RT regions were not influenced by subtype-specific characteristics (Supplementary Fig. 7B–D).

### Evolutionary timing of ART within individual tumours

The above analysis focused on aggregated mutations across all LUAD and BRCA tumours respectively which may mask important inter-tumour heterogeneity. Therefore, we next evaluated the extent to which ART may correlate with mutation acquisition between individual tumours. We first performed a bootstrapping test to identify tumours with a significant difference (bootstrap *p*-value < 0.001) in mutation density between Early_N+T_ and Late_N+T_ replicated regions. We reasoned these tumours harbour a sufficiently high mutation burden to evaluate whether ART also has an association with mutation density (“Methods” section). We identified a significant difference in 178 out of 470 LUAD tumours, whereas only 3 out of 482 BRCA tumours exhibited a significant difference (Supplementary Fig. 7E), consistent with the significantly lower mutation burden in BRCA tumours. Thus, only these 178 LUAD tumours were included for the per-tumour analysis.

When comparing the average burden of mutations that were present in the MRCA between different unaltered RT and ART regions for each of the 178 LUAD tumours, each tumour presented a clear difference in the mutation distribution in ART versus unaltered RT regions (Fig. 3C, D), consistent with our observations at the cohort level (Fig. 3B). These results support the notion that shared ART regions identified in different cancer cell lines (Fig. 2E) within the same cancer type can be used as a proxy for ART present in human tumours. Analysing the fraction of mutations accumulated in the MRCA prior to ART within each tumour (Early_N_-to-Late_T_ range 7%–95%; Late_N_-to-Early_T_ range 0.1%–65%) revealed that in all analysed tumours a small fraction of truncal mutations likely accumulated after ART, which suggests that ART occurs prior to the emergence of MRCA in LUAD tumour evolution (Fig. 3D). However, it is notable that in 15% (26/178) of LUAD tumours we observed that the Early_N_-to-Late_T_ ART change potentially occurred prior to the Late_N_-to-Early_T_ change.

To further exclude the potential bias caused by inter-tumour heterogeneity and provide additional validation of the association between ART and mutation accumulation across the genome, we performed Repli-seq and WGS for two patient-derived cell lines (PDCs; CRUK0557-CL and CRUK0977-CL). These cultures were derived from two patients with diagnoses of LUAD enrolled in the TRACERx study (“Methods” section). Similar to other LUAD cell lines, 5.7% of the CRUK0557-CL genome and 6.0% of the CRUK0977-CL genome had ART relative to the T2P cell line, which was used as a tissue-of-origin control (Fig. 3E; “Methods” section). Consistent with ART influencing the acquisition of mutations, we observed a significantly higher mutation burden in genomic regions that were replicated late in cancer but early in normal compared to genomic regions that were replicated early in cancer but late in normal. Estimating the fraction of mutations that likely accumulated prior to ART within the two PDCs confirmed that ART is likely to be an early event during cancer evolution in LUAD (Early_N_-to-Late_T_: 10% and 26%; Late_N_-to-Early_T_: 11% and 12%; Fig. 3F). This observation supports our conclusion that ART occurs early during tumour evolution and that the shared ART regions identified in LUAD cell lines can be used to explore the impact of ART on mutation accumulation in larger cohorts of patient tumours.

### Differential correlation of ART and chromatin structure with mutation accumulation

**Fig. 4:**
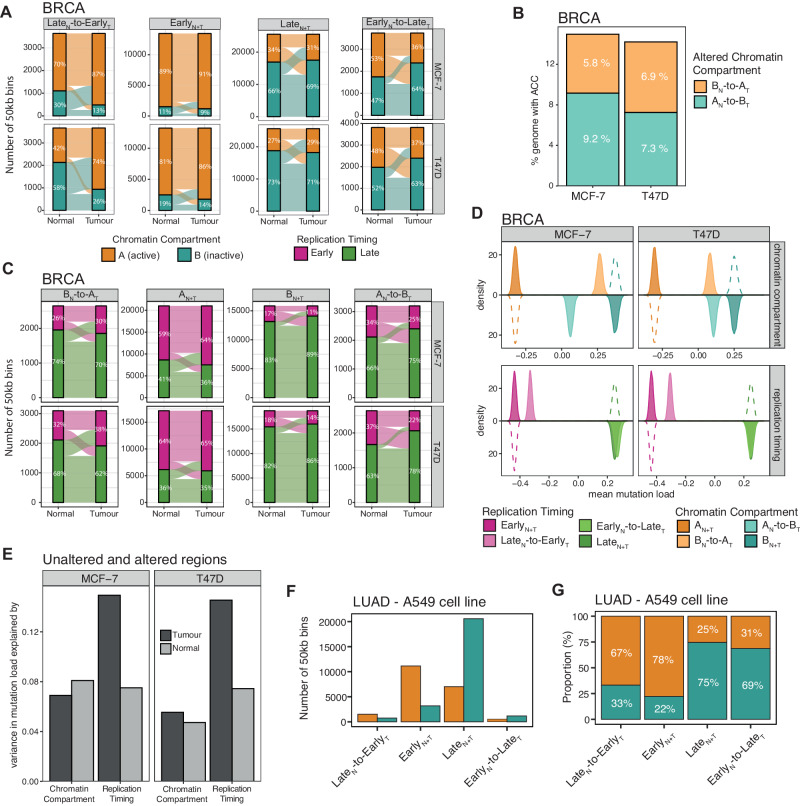
Alterations to replication timing and chromatin structure in cancer. **A** Alluvial plots highlighting the difference in the fraction of genomic bins located in the A compartment and B compartment in unaltered replication timing (RT) and altered replication timing (ART) regions for the two breast carcinoma (BRCA) cell lines MCF-7 (tumour) and T47D (tumour) compared to the cell line derived from their likely tissue-of-origin HMEC (non-malignant as “normal”). **B** The fraction of the genome exhibiting altered chromatin compartment (ACC) regions in two BRCA cell lines (MCF-7 and T47D) relative to HMEC. **C** Alluvial plots highlighting the difference in the fraction of genomic bins presenting early and late RT in unaltered chromatin compartment and ACC regions for the two BRCA cell lines MCF-7 (tumour) and T47D (tumour) compared to HMEC (non-malignant as “normal”). **D** The top panels show the distribution of bootstrapped mean mutation load values in BRCA tumours in unaltered chromatin compartments and ACC regions. The bottom panels show the distribution of bootstrapped mean mutation load values in BRCA tumours in unaltered RT and ART regions. **E** Variance in BRCA mutation load explained by chromatin or replication timing signal in normal (HMEC) and tumour (MCF-7 or T47D). The bars represent the *R*^2^ values derived from separate univariate linear models with mutation load as an independent variable and the chromatin signal or RT signal in normal or cancer cells as a dependent variable. Notably, RT in tumours can explain more of the variance in mutation load than the other factors. **F**, **G** The counts (**F**) and proportions (**G**) of differently replicated genomic bins classified as A or B compartment in the LUAD cell line A459.

In order to elucidate the relative associations between alterations to replication timing and chromatin localisation, and mutation distribution, we analysed publicly available Hi-C data (“Methods” section). Genomic regions in 50 kb bins were classified as A compartment (active genomic regions with an open chromatin structure and a location at the interior of the nucleus) or B compartment (inactive genomic regions with a closed chromatin structure and a location at the periphery of the nucleus) in cell lines for which we also had performed Repli-seq analyses (*i.e*., HMEC, MCF-7, T47D and A549). As expected, unaltered Early_N+T_ replicated regions were located preferentially in the A compartment while Late_N+T_ were located preferentially in the B compartment. However, we also observed a significant increase in the fraction of genomic bins classified as A compartment among Late_N_-to-Early_T_ replicated regions in two BRCA cell lines (MCF-7 and T47D) compared to their normal reference HMEC (Fig. 4A). Consistently, the fraction of genomic bins identified as B compartment was significantly increased among Early_N_-to-Late_T_ regions in both BRCA cell lines compared to their normal reference (Fig. 4A).

We next quantified the degree of altered chromatin compartment (ACC) regions across the genome in BRCA cells relative to their normal reference. 50 kb bins defined as A compartment in normal but B compartment in cancer cells were classified as altered A_N_-to-B_T_ regions whereas bins within the B compartment in normal but A compartment in cancer cells were labelled altered B_N_-to-A_T_ regions. Unaltered chromatin compartment regions were classified as either unaltered A_N+T_ or B_N+T_ regions (“Methods” section).

We observed a similar fraction of genomic bins with ACC to those with ART (A_N_-to-B_T_ average 8.3%, B_N_-to-A_T_ average 6.4%; Fig. 4B). As expected, unaltered A_N+T_ regions were enriched in 50 kb genomic bins predominantly exhibiting early RT and B_N+T_ regions were associated with late RT in both normal and cancer cells (Fig. 4C). A higher fraction of bins replicated early in cancer than replicated early in normal was observed among B_N_-to-A_T_ compartment regions and a higher fraction of bins replicated late in cancer than replicated late in normal was detected in A_N_-to-B_T_ regions (Fig. 4C), highlighting the relationship between RT and chromatin structure even in altered regions in cancer.

To explore the association between ACC and mutation accumulation, we applied a bootstrapping approach to compare the average load of mutations that were present within the MRCA between different unaltered chromatin compartments and ACC regions (“Methods” section). While the ACC genomic regions were associated with a significant change in mutation density, the observed differences appeared less pronounced compared to the association with ART (Figs. 4D; 3B). In fact, the variability in local mutation burden across the genome in BRCA tumours was better predicted by the RT signal in cancer cells than by the chromatin compartment signal in cancer or any of the two signals in normal cells (Fig. 4E; Supplementary Fig. 7F; “Methods” section). These data suggest that in BRCA, ART may have a stronger correlation with mutation accumulation than alterations to the chromatin landscape.

In the absence of publicly available Hi-C data for paired normal and LUAD cells, we assessed the fraction of A and B compartment regions among unaltered RT and ART regions in A549 cells. We observed an increase in bins classified as A compartment in Late_N_-to-Early_T_ compared to unaltered Late_N+T_ regions and an increase of B compartment bins in Early_N_-to-Late_T_ compared to unaltered Early_N+T_ regions in LUAD cells, supporting an association between ART and ACC in LUAD (Fig. 4F–G).

### The interplay between ART and mutational processes

The non-uniform distribution of mutations across the genome is influenced by different activities of DNA damage and repair mechanisms^5,31^. Differences in DNA damage can be caused by various mutational processes inducing specific mutation patterns, termed mutational signatures^32^. The exposure of mutational signatures across the genome in relation to different epigenetic features including RT, has revealed differences in the number of mutations induced by certain mutational processes in early versus late replicated genomic regions^33,34^.

**Fig. 5:**
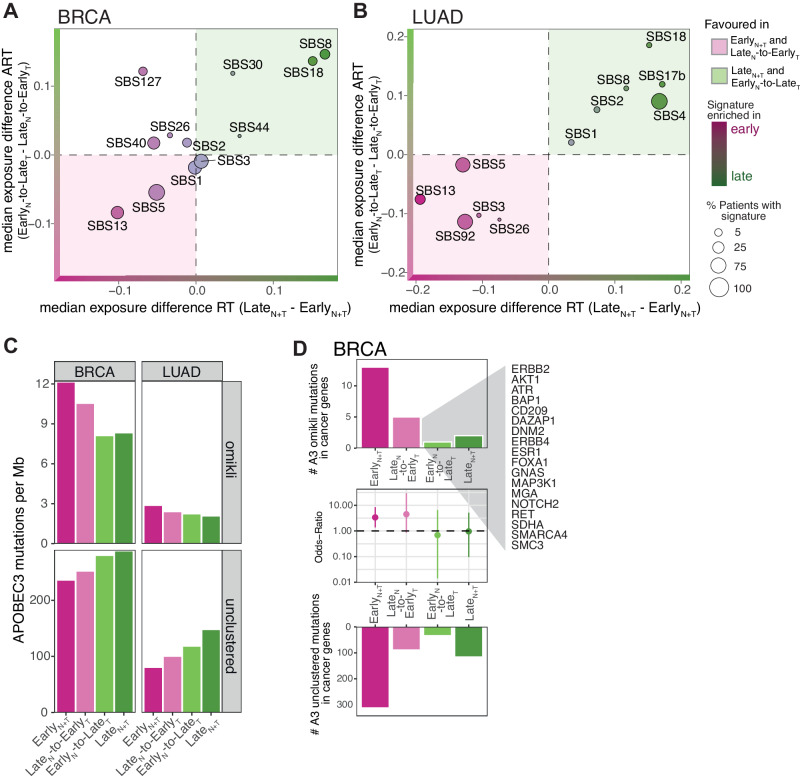
The correlation of ART with the activity of DNA damage and repair mechanisms. **A**, **B** Scatter plot comparing the median difference in the exposure of mutational signatures in unaltered Late_N+T_ and Early_N+T_ replicated regions (x-axis) against the median difference of mutational signature exposures in altered Late_N_-to-Early_T_ and Early_N_-to-Late_T_ replicated regions (y-axis) in breast carcinoma (BRCA) (**A**) and lung adenocarcinoma (LUAD) (**B**) tumours. Signatures located in the top right quadrant were found to be enriched in Late_N+T_ and Early_N_-to-Late_T_ replicated regions. Signatures located in the bottom left quadrant were found to be enriched in Early_N+T_ and Late_N_-to-Early_T_ replicated regions. The size of the points demonstrates the fraction of tumours in which the different mutational signatures have been found active. **C** The number of APOBEC3-mediated omikli mutations (top bar plots) and the unclustered APOBEC3 mutations (bottom bar plots) per Mb in different unaltered replication timing (RT) and altered replication timing (ART) regions in BRCA and LUAD tumours. **D** The number of APOBEC3 mutations per Mb in an omikli (top) and unclustered (bottom) context in cancer-associated genes localised at different unaltered RT and ART regions in BRCA tumours. Middle plot: the odds ratio is shown as dots and the 95% confidence intervals as vertical lines obtained by Fisher’s tests to investigate whether there was a significant enrichment of APOBEC3-mediated omikli mutations in cancer genes relative to unclustered APOBEC3 mutations in different unaltered RT and ART regions. Cancer-associated genes with Late_N_-to-Early_T_ replication timing in BRCA are highlighted.

To explore whether the distribution of mutations induced by different biological processes across the genome was impacted by changes to the RT programme, we performed a de novo mutational signature extraction analysis using the whole-genome sequencing data from the 952 BRCA and LUAD tumours with a hierarchical Dirichlet process (HDP) model in relation to the different RT regions^35,36^ (“Methods” section). We identified 13 known mutational signatures in BRCA and 12 in LUAD tumours (Supplementary Fig. 9). The activity of mutational processes was similar between unaltered Early_N+T_ and Late_N_-to-Early_T_ replicated regions and also between unaltered Late_N+T_ and Early_N_-to-Late_T_ replicated regions in BRCA and LUAD (Fig. 5A, B, Supplementary Fig. 10A, B). This suggests that the majority of mutations which were accumulated as a result of these mutational processes likely occurred after ART.

In LUAD, two smoking-associated signatures (SBS4 and SBS92) were detected. While SBS4 was enriched in Late_N+T_ and Early_N_-to-Late_T_ regions, SBS92 was enriched in Early_N+T_ and Late_N_-to-Early_T_ regions (Fig. 5B). This finding suggests that these two smoking-associated signatures may reflect two different biological processes related to the mutagenic insults of smoking. Given that the burden of SBS4 mutations in LUAD has previously been found to be highly correlated with smoking history, as measured by pack years^37^, the observation that smoking-induced mutations were also enriched in Early_N_-to-Late_T_ replicated regions suggests that the patients were still smoking following the change in RT. This is consistent with the notion that ART is a relatively early event during LUAD evolution. In BRCA, SBS127, which has recently been reported to be present in the majority of BRCA tumours but whose aetiology is still unknown^38^, was identified to be enriched in Early_N+T_ and Early_N_-to-Late_T_ replicated regions. This suggests that the corresponding mutagenic process was potentially active before ART and thus may indicate one of the earliest mutagenic processes during BRCA evolution (Fig. 5A).

Taken together, these results support the hypothesis that ART represents an early evolutionary event during BRCA and LUAD development. Furthermore, the link between ART and mutational signatures suggests that a shift in RT correlates with the likelihood of mutation acquisition in different genomic regions.

### ART and the activity of DNA mismatch repair

Mutations induced by the APOBEC3 family of cytidine deaminases have been detected in tumours deriving from many different cancer types^34,39–41^. We observed that the APOBEC3-related mutational signature SBS13 was enriched not only in unaltered Early_N+T_ replicated regions but also in altered Late_N_-to-Early_T_ replicated regions in BRCA and LUAD (Fig. 5A, B). A recent study found that APOBEC3-mediated mutations in early replicated regions often occur in small mutation clusters (2–4 clustered mutations) termed omikli events, which are likely promoted by the enriched DNA mismatch repair (MMR) activity in early replicated regions^42^.

To elucidate whether changes to the RT programme during malignant transformation also have the potential to change the activity of MMR, we applied the hyperClust method^42^ to identify omikli events and differentiated them from unclustered mutations within or outside an APOBEC3 context (“Methods” section). Consistent with previous work, we found a significant enrichment of APOBEC3-mediated omikli events in Early_N+T_ regions in comparison to Late_N+T_ regions (Fig. 5C). Moreover, by leveraging our ART data, we uncovered an enrichment of APOBEC3-mediated omikli events in Late_N_-to-Early_T_ compared to Late_N+T_ replicated regions, which suggests that the activity of MMR was deregulated after the RT shift occurred in these regions (Fig. 5C, Supplementary Fig. 10C, D).

Given that MMR is directed towards genes in early replicated regions that are crucial for essential functions of the cell, mutagenic processes induced by MMR activities have been associated with a high likelihood of inducing driver mutations during tumour evolution^42,43^. We identified a significant enrichment of APOBEC3-mediated omikli mutations in cancer-associated genes in unaltered Early_N+T_ (Fisher’s test: odds ratio = 3.38, *p*-value = 0.004) as well as in Late_N_-to-Early_T_ (Fisher’s test: odds ratio = 4.49, *p*-value = 0.041) replicated regions in BRCA (Fig. 5D). A total of 18 cancer genes including *ERBB2/HER2, ATR, ESR1* and *MAP3K1* were identified to be Late_N_-to-Early_T_ replicated in BRCA and affected by an APOBEC3-mediated omikli event in at least one tumour in the breast cancer cohort. These results suggest that ART has the potential to increase the likelihood of acquiring specific driver mutations, which might lead to a fitness advantage of emerging subclones. This analysis could not be applied to the LUAD cohort as the numbers of APOBEC3-mediated omikli mutations in cancer genes within ART regions were too low.

Taken together, changes in the RT programme during malignant transformation were found to correlate with the activity of mutagenic processes and MMR.

### Late_N_-to-Early_T_ replicated regions are associated with increased transcription

**Fig. 6:**
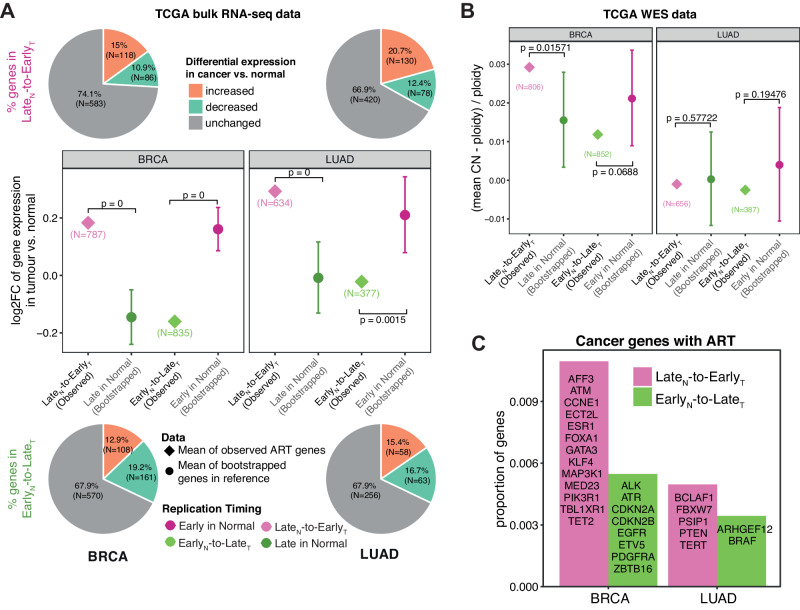
The genomic and transcriptomic features of ART regions in BRCA and LUAD. **A** Comparison of the mean log2 fold change (log2FC) of Late_N_-to-Early_T_ replicated genes (787 genes in BRCA and 634 genes in LUAD) versus 100,000 times randomly selected (bootstrapped) late replicated genes in normal cells, and the equivalent comparison between Early_N_-to-Late_T_ replicated genes (835 genes in BRCA and 377 genes in LUAD) versus bootstrapped early replicated genes in normal cells. Proportions of differentially expressed genes are displayed as pie charts with the numbers and proportions of genes included in each group annotated accordingly. The observed mean log2FC are presented as diamonds in the plot in the middle while the bootstrapped results are shown as dots with error bars. The error bars represent the 95th percentile of the bootstrapped mean log2FC values. **B** Comparison of the mean copy number values relative to tumour ploidy of Late_N_-to-Early_T_ replicated genes (806 genes in BRCA and 656 genes in LUAD) in cancer cells versus bootstrapped late replicated genes in normal cells, and equivalently the comparison between Early_N_-to-Late_T_ replicated genes (852 genes in BRCA and 387 genes in LUAD) in cancer cells versus bootstrapped early replicated genes in normal cells. The observed values are presented as diamonds while the bootstrapped results are shown as dots with error bars. The error bars represent the 95th percentile of the bootstrapped mean copy number values relative to tumour ploidy. In **A**, **B**, the annotated *p*-values represent the empirical *p*-values which were calculated by counting how many bootstrapped mean log2FC values of Late_N+T_ genes were greater than the observed mean values of Late_N_-to-Early_T_ genes divided by the total number of iterations, or equivalently, how many bootstrapped mean log2FC values of Early_N+T_ genes were lower than the observed mean values of Early_N_-to-Late_T_ genes divided by the total number of iterations. **C** Cancer-associated genes identified in altered replication timing (ART) regions in breast carcinoma (BRCA) and lung adenocarcinoma (LUAD).

Genomic alterations reflect footprints of mutational processes that have been active during tumour development. To further investigate whether ART correlates with the phenotype of cancer cells at the time of tumour resection, we investigated the association between ART and gene transcriptional levels in tumours. We first calculated a differential gene expression score (log2-transformed fold change (log2FC)) for each expressed gene in tumour samples compared to the paired normal tissues from TCGA using their bulk RNA sequencing (RNA-seq) data (“Methods” section). Comparing the differential expression score of genes with ART versus those with unaltered RT in BRCA and LUAD revealed that Late_N_-to-Early_T_ replicated genes were frequently overexpressed compared to matched normal samples, whereas the opposite was true for Early_N_-to-Late_T_ genes (Pie plots in Fig. 6A), supporting previous findings in prostate cancer and leukaemia^4,16^.

To explore the relationship between transcription and RT, we compared genes in regions of ART to their unaltered counterparts, using a bootstrapping approach to control for gene number (“Methods” section). We observed that Late_N_-to-Early_T_ replicated genes in cancer cells harboured higher expression levels compared to other unaltered Late_N+T_ genes both in BRCA and LUAD (Dot plots in Fig. 6A). Thus, a change in RT during malignant transformation correlates with the expression levels of affected genes. Conversely, the expression of Early_N_-to-Late_T_ genes was lower than unaltered Early_N+T_ genes (Dot plots in Fig. 6A). When we applied a similar bootstrapping test to the copy number data in TCGA tumours, we observed no clear association between ART and copy number gains or losses, suggesting that the changes in expression of genes with ART were independent of copy number alterations (Fig. 6B; “Methods” section). Furthermore we discovered cancer genes to be affected by ART in BRCA and LUAD (Fig. 6C), supporting our previous findings that ART plays an important role during malignant transformation.

## Discussion

Prior work has documented that the RT programme correlates with the burden of genomic^5,6,12,28,29,33^, transcriptomic^44,45^ and epigenetic^16^ alterations in cancer. Although the contribution of RT to the acquisition of mutational signatures has been studied in several tumour types^46^, the extent to which the RT programme changes during cancer development and the relationship of this with mutation acquisition has not been comprehensively explored. Here, we investigated RT in multiple BRCA and LUAD cancer cells and compared these to non-malignant cells derived from the likely tissue-of-origin, to elucidate the extent and importance of altered replication timing (ART) in tumour evolution.

We integrated the whole-genome and transcriptome sequencing data of breast and lung tumours and revealed that alterations in the RT programme in cancer correlate significantly with both the acquisition of mutations and gene expression across the cancer genome. Our analyses of mutation accumulation in ART and unaltered RT regions suggest that changes to the RT programme occur relatively early during the development of both BRCA and LUAD, reshaping their genomic landscape and playing a role in their evolutionary trajectories. Further analyses, incorporating data from pre-invasive disease, may enable an exploration of whether this change occurs prior to malignant transformation, and whether it could be exploited for early detection of cancer.

Our data suggest that cancer cells maintain the same ratio of early to late replicated regions across the genome as observed in non-malignant cells, with 1/3 of the genomic regions being early replicated and 2/3 being late replicated (Supplementary Fig. 3). The maintenance of this ratio might be related to the limited resources for DNA replication in early versus late S phase, the limited number of dormant replication origins to complete DNA replication under replication stress or the limited space in the interior of the nucleus to harbour highly transcribed and early replicated euchromatin with an open structure^47,48^. This suggests that Late_N_-to-Early_T_ changes may occur first as a response to DNA replication stress, which is then followed by Early_N_-to-Late_T_, consistent with our data timing the ART in cancer.

The RT in human cells has been fine-tuned as a result of natural selection. It is therefore tempting to speculate that shifts in RT during cancer development represent the footprint of selection moulding the cancer genome. For instance, alterations in RT may protect certain genomic regions from DNA damage and replication stress which is pervasive in lung and breast cancer evolution.

Despite the small proportion of the genome presenting ART from normal to cancer cells, we observed that ART regions play an important role in cancer evolution by shaping the mutation landscape across the genome and enriching omikli events in Late_N_-to-Early_T_ regions which are prone to cause cancer driver events. A large proportion of Early_N_-to-Late_T_ regions were classified as B compartment in both normal and cancer cells while many Late_N_-to-Early_T_ regions were classified as A compartment (Fig. 4A). Although this observation might suggest that some ART regions have already been poised to change from normal to cancer, we still observe significant differences between altered replication timing (ART) and altered chromatin compartment (ACC) regions across the genome, especially when assessing differences in their correlations with mutation density.

Moreover, we also discovered a correlation between increased gene expression and Late_N_-to-Early_T_ replication timing, but the causal link between these two has been controversial in the literature^3,17–19^. The interplay among changes in replication timing, chromatin architecture and gene expression is complicated and may reflect multiple biological processes, such as transcription-replication conflicts, limited resources of replication origins, and distinct stimuli of replication stress among others. Further studies are warranted to unveil the causal links between these three factors leading to genomic instability, malignant transformation and cancer evolution.

This study is not without limitations. Our work highlighted that copy number gains and losses can confound the interpretation of mutation acquisition across the genome, and while our study attempts to correct for this, with more sophisticated sequencing technologies enabling phasing or variants with individual alleles, it will be possible to more accurately resolve the timing of mutations and link these to copy number alterations. Furthermore, we focused on recurrent ART regions identified in multiple cancer cell lines, enabling us to translate our analysis from cancer cells to patient tumours. One caveat of our approach is that we used a restricted sample size to define ART and only included one optimal reference cell line each for LUAD and BRCA tumours, thus neglecting any potential heterogeneity among different normal cells. Furthermore, there may be some additional impact of long-term cell culturing and immortalisation which could also influence our analyses^49^. Moreover, while our data point towards consistent patterns of ART across tumours, as evidenced by a consistent association between ART and mutation acquisition across LUAD tumours, a significant overlap of ART between cell lines, and a similar pattern observed when restricting to PDCs, there will likely be both intra- and inter-tumour heterogeneity that is neglected by our approach. Conceivably, an exploration of the intra-tumour heterogeneity of the RT programme and its relationship with genomic alterations in individual patient tumours may be possible using single-cell sequencing data, which is an area of current research^50^. Nevertheless, our work provides a comprehensive replication timing dataset of lung and breast cancer cells together with their matched optimal tissue-of-origin and offers an insight into the interplay between ART and cancer development.

In conclusion, our integrated data analysis supports the crucial role of ART in shaping the genomic and transcriptomic landscape in breast and lung cancer as an early truncal event during tumour evolution.

## Methods

This study complies with all relevant ethical regulations required by the University College London Cancer Institute and the Francis Crick Institute.

### Whole-genome sequencing cohorts

#### The Genomics England Limited (GEL) lung cohort of the 100,000 Genomes Project

We used the whole-genome sequencing data of lung adenocarcinoma (LUAD) tumours from the Genomics England Limited version 8 cohort of the 100,000 Genomes Project^21^. The GEL version 8 dataset can be accessed via https://www.genomicsengland.co.uk/about-gecip/for-gecip-members/data-and-data-access.

After multiple steps of quality control (QC), 470 LUAD tumours were included in this study (from 259 female patients and 211 male patients). We excluded formalin-fixed paraffin-embedded samples (FFPE) and low-purity samples that failed copy number or structural variant calling. Some samples were duplicated with discordant information in the cancer summary table provided by GEL and were excluded from the final cohort.

##### Correction for reference bias

The Illumina Isaac pipeline^51^ has been used in the 100,000 Genomes Project to align and process the whole-genome sequencing data to the hg38 assembly. However, recent studies have demonstrated that the soft clipping of semi-aligned reads performed by the Isaac aligner leads to a reference bias which affects the calling of somatic copy number alterations (SCNAs) as well as purity and cancer cell fraction (CCF) estimations in cancer^52^. To address this caveat, a tool called fixVAF^52^ was developed by Cornish et al. (https://github.com/danchubb/FixVAF) to remove sources of reference bias ensuring a robust CCF estimation. We applied fixVAF to the BAM files and VCF files of the GEL lung cohort which were produced by the Genomics England core pipeline.

##### Variant calling

As part of the Genomics England core pipeline, Strelka^53^ has been applied for somatic variant calling. The resulting VCF files were corrected for biases in the variant allele frequency (VAF) by applying fixVAF^52^ which includes multiple filtering and QC steps. Additional filters for single nucleotide variants (SNVs) and INDELs, informed by the TRACERx pipeline^54^, were applied. This includes that any variant located within a blacklist region of the genome, as used in the TRACERx pipeline and informed by the “blacklisted” regions reported on ENCODE^55^, were removed.

Additional filters that an SNV had to pass to be included in the final mutation table:

- VAF ≥5%
- alternative reads ≥5
- Germline VAF <1%
- Germline number of alternative reads <5
- Total depth ≥30

Additional filters that an INDEL had to pass to be included in the final mutation table:

- VAF ≥5%
- alternative reads ≥10
- Germline VAF <1%
- Germline number of alternative reads <5
- Total depth ≥50

##### Copy number calling

We have applied Battenberg^56^ (https://github.com/Wedge-lab/battenberg) to the DNA sequencing data for the estimation of the copy number profile, ploidy and purity of the lung tumours. For this a nextflow pipeline^57^ was developed using the fixVAF corrected tumour and normal BAM files as well as the corrected VCF file per tumour as input. As a first step of the pipeline, the initial profiling of copy number alterations was conducted using Battenberg. Afterwards, multiple assessment steps were applied to evaluate the estimated profile. If any of these criteria were not met, the sample was re-processed up to 4 times using an updated purity estimate. The quality assessment included the evaluation of the concordance of the copy number profile with the VAF distribution of the mutations. The sample failed if the absolute difference between the sample purity computed by Battenberg and the VAF estimated purity was >5%. Furthermore, the correct calling of whole-genome doubling (WGD) was evaluated. If >30% of the genome presented an average total copy number state of about 0.5 or 1.5, it was assumed that Battenberg had incorrectly not called WGD. Also, if >20% of the genome presented a copy number state 2:2 (tetraploid) or 3:3, and <10% of the genome presented an odd copy number state, and no peak corresponding to a multiplicity of 1 was observed in the VAF distribution of SNVs in 2:2 (tetraploid) regions, then it was assumed that Battenberg had incorrectly called WGD. The clonal architecture was characterised using DPClust to assess the presence of a clonal mutation cluster consisting of at least 5% of all variants with a CCF > 0.9 and <1.1. A sample failed if a “super-clonal” cluster could be identified by DPClust which contains at least 5% of all variants with a CCF > 1.1. Furthermore, a sample failed if it presented large clonal or subclonal homozygous deletions >10 Mb. If a sample passed all the criteria mentioned above the profile of clonal and subclonal copy number alterations as well as the purity and ploidy estimate were returned. If a sample failed any of the criteria the purity was re-computed using the peaks of the VAF distribution. The re-computed purity was used to re-estimate the copy number profile and the quality assessment steps were applied again. This process was repeated 4 times in total. If after the fourth time, the sample still failed any of the criteria, it was marked as failed. In total, 1531 lung cancer tumours including multiple different histologies were run through the pipeline with 1119 of them marked as passed and 413 failed. Afterwards, all failed and passed samples were manually reviewed. This resulted in 37/1119 originally passed samples to fail and 70/413 failed samples to pass. In total, 1152 samples passed and 380 samples failed copy number QC.

Applying multiple exclusion criteria as mentioned above resulted in a total GEL lung cohort of 1027 tumours of which 470 were lung adenocarcinomas.

#### The 560 breast cancer whole-genome cohort

Mutation and copy number calls for the 560 breast cancer WGS cohort were provided by the publication “Landscape of somatic mutations in 560 breast cancer whole-genome sequences” by Nik-Zainal et al.^22^. This data was aligned to the hg19 assembly. Only tumours for which somatic variants and copy number profiles were provided were used in this study. In the literature the human mammary epithelial cells (HMEC) have been reported to be the originating tissue for lobular and ductal breast cancer subtypes^26^, hence only tumours with this subtype were included in this analysis. This resulted in a total cohort of 482 breast cancer tumours (from 479 female patients and 3 male patients).

### Publicly available datasets

#### The Cancer Genome Atlas (TCGA) data

Gene expression and copy number calls for BRCA and LUAD tumours generated by The Cancer Genome Atlas pilot project established by the NCI and the National Human Genome Research Institute were downloaded. The data were retrieved through database of Genotypes and Phenotypes (dbGaP) authorisation (accession no. phs000854.v3.p8). Information about TCGA and the investigators and institutions who constitute the TCGA research network can be found at https://cancergenome.nih.gov/. Raw read counts were downloaded for 830 BRCA (ductal and lobular) tumours and 517 LUAD tumours to identify expressed genes in each cancer type. For 149 of these tumours (91 BRCA and 58 LUAD), RNA-seq data for their adjacent normal tissues were available. These 149 paired normal and tumour samples were used for the differential expression analysis. ASCAT^58^ initiated copy number profiles were downloaded for 766 BRCA (ductal and lobular) and 708 LUAD tumours to evaluate whether differences in gene expression were driven by copy number alterations in the tumour.

#### Repli-seq data on ENCODE

The replication-timing sequencing (Repli-seq) data in form of fastq-files for 16 cell lines were downloaded from ENCODE^23,24^ (Caki2, NCI-H460, A549, T47D, SK-N-MC, BG02, HeLa-S3, HUVEC, SK-N-SH,HepG2, IMR-90, BJ, G401, LNCAP, keratinocyte, MCF-7). The accession numbers of the files are reported in Supplementary Table 2. This data was provided by two different research groups, David Gilbert (FSU) and John Stamatoyannopoulos (UW). Both groups applied different Repli-seq assays resulting in different number of time points analysed during S phase. While David Gilbert’s group used a similar assay to ours, their Repli-seq data included fastq-files for early and late S phase reads (Caki2, NCI-H460, A549, T47D, SK-N-MC, G401, LNCAP). The Repli-seq data of the other cell lines covered 6 time points instead of 2 including G1, S1, S2, S3, S4 and G2. In order to make the data comparable the fastq-files containing G1, S1 and S2 reads were merged together after alignment and used as early replicated reads for further analyses whereas the resulting bam files of the fastq-files containing S3, S4 and G2 reads were merged and used as late replicated reads.

#### Hi-C data on ENCODE

The ENCODE portal^23,24^ was used to identify BRCA and LUAD samples that provided preprocessed Hi-C data that overlapped with the samples used in our RT analysis (HMEC, MCF-7, T47D, A549). This Hi-C data was created with two different experimental assays; intact Hi-C and in situ Hi-C. While in situ Hi-C data was available for A549 and T47D, intact Hi-C data was provided for MCF-7 and both types were available for HMEC. The accession numbers of the samples and files are shown in Supplementary Table 3.

To identify the nuclear compartments, we used POSSUM^59^ specifying “-n SCALE” normalisation and 50 kb output resolution to match the interval size used for the RT analysis. In downstream analyses, a negative value is interpreted as a B compartment and a positive value as an A compartment. The BEDTools^60^ version 2.3.0 intersect function was used to attribute nuclear compartments to RT intervals.

#### Genomic and transcriptomic data of cell lines

The gene expression data and mutation profiles for all cancer cell lines were downloaded from DepMap (https://depmap.org/portal/). Copy number data for all cancer cell lines, except for SK-BR3, were downloaded from the Catalogue of Somatic Mutations in Cancer (COSMIC). The copy number profiles were identified by applying PICNIC^61^ to the Affymetrix SNP6.0 array data for each cell line.

#### Cancer gene lists extracted from publicly available data

Cancer driver genes for lung and breast cancer were extracted from publicly available datasets. Mutational breast cancer driver genes reported by Bailey et al.^62^, Martincorena et al.^1^, Nik-Zainal et al.^22^, and published on the intOGen website (https://www.intogen.org/search)^63^ were used in this analysis. Lung cancer driver genes identified in the TRACERx 100 cohort^54^, the TRACERx 421 cohort^64^, Bailey et al.^62^, Martincorena et al.^1^ and Berger et al.^65^ were summarised and used in this analysis. Oncogenes and tumour suppressor genes were downloaded from COSMIC census gene lists (https://cancer.sanger.ac.uk/census)^66^.

### Cell culture

All cell lines from lung and breast tissues applied in this study (Supplementary Table 1) were incubated at 37 °C with 5% CO_2_, according to the lab standard protocols. A total of 11 lung and 4 breast cell lines were included, which were related to breast cancer (BRCA), lung adenocarcinoma (LUAD) and lung squamous cell carcinoma (LUSC).

The pulmonary alveolar epithelial type II cells (T2P) and the other six lung cancer cell lines (A549, H1650, H1792, H2009, H520 and H2170) which were provided by Cell Services at the Francis Crick Institute, were maintained in RPMI medium (Thermo Fisher; 21875034) supplemented with 10% heat-inactivated foetal bovine serum (FBS) (v/v; 10082-147), 1× penicillin–streptomycin (100 U/ml penicillin, 100 μg/ml streptomycin; Gibco; 15070) and 1x L-Glutamine (v/v). The lung cell line SW900 was purchased from ATCC^67^ and cultured in ATCC-formulated Leibovitz’s L-15 Medium (ATCC; 30-2008) supplemented with 10% FBS. The TT1 (pulmonary alveolar epithelial type I cells) cell line^68^ was obtained from Dr Michele Chiappi and Professor Terry Tetley (National Heart and Lung Institute, Imperial College London, UK). Immortalised TT1 cells were derived from human primary pulmonary alveolar epithelial type II cells but have a phenotype resembling alveolar epithelial type I cells^68^. TT1 was cultured in DCCM-1 (Geneflow Ltd, K1-0502) supplemented with 10% penicillin–streptomycin–glutamine (Thermo Fisher, 10378016) and 10% NCS (new-born calf serum, heat inactivated, New Zealand origin; 26010074).

Breast cell lines, including human mammary epithelial cells (HMEC, known as hTERT-HME 1 cell line), MCF10A, SK-BR3 and MDA453, were provided by Cell Services at the Francis Crick Institute. Both HMEC and MCF10A are considered normal, non-malignant, immortalised breast cell lines. However, MCF10A cells were actually derived from fibrocystic breast disease and display characteristics of luminal ductal cells, rather than mammary epithelial cells. Given that breast cancer cell lines involved in our study are mammary gland epithelial carcinoma, HMEC cell line, rather than MCF10A, is considered to represent the originating tissue-of-origin of BRCA.

HMEC was cultured in HMEC-Mammary Epithelial MEGM (Lonza, CC-2551) supplemented with MEGM BulletKit (Lonza, CC-3150) and ReagentPack Subculture Reagents (Lonza, CC-5034). MCF10A cells were cultured in DMEM/F12 media (Invitrogen) supplemented with 5% horse serum (Invitrogen #16050-122), 20 ng/ml human epidermal growth factor (hEGF) (Peprotech, AF-100-15), 0.5 µg/ml hydrocortisone (Sigma #H-0888), 100 ng/ml cholera toxin (Sigma #C-8052), 10 µg/ml insulin (Sigma #I-1882), and 1× antibiotics (Invitrogen #15070-063). SK-BR3 was cultured in ATCC-formulated McCoy’s 5a Medium Modified (30-2007) supplemented with 10% of FBS. MDA453 cells were cultured in ATCC-formulated Leibovitz’s L-15 Medium (30-2008) supplemented with 10% of FBS.

For Cell Authentication, we use STR (Short Tandem Repeat) Profiling for all our human cell lines using the Promega PowerPlex16HS system. This profile is compared back to any available commercial cell banks (such as ATCC). We confirm the species is correct using a primer system based on the Cytochrome C Oxidase Subunit 1 gene from mitochondria – we call this test Species ID. Cell Authentication is carried out in house within the Francis Crick Institute. For Mycoplasma screening we primarily use two different tests – Agar Culture (which involves culturing any mycoplasma that may be present in the cell culture on specialised agar) and Fluorescent staining using the Hoescht Stain. A third detection method, the PCR mycoplasma test (ATCC), is used on occasion when a rapid result is required. Mycoplasma testing is carried out in house routinely within the Francis Crick Institute and has been negative for cell lines used in this study.

### Patient-derived cell cultures

Primary tumour cell cultures were isolated from two patients (CRUK0977, female and CRUK0557, male) diagnosed with LUAD within the lung TRACERx study with informed consent, sponsored by University College London (UCL/12/0279) and has been approved by an independent Research Ethics Committee entitled “the Health Research Authority NRES Committee London - Camden & Islington” (13/LO/1546). The methods and details of the ethical review have been published here (10.1002/ijc.31383)^69^. The isolation and expansion of the CRUK0557 cell line (CRUK0557-CL) was as previously described^69^ except that cells were cultured in the absence of mouse 3T3-J2 fibroblast feeder cells prior to DNA extraction. The CRUK0977 cell line (CRUK0977-CL) was isolated by the culture of primary tumour tissue in RPMI-1640 medium containing 10% FBS, 1 mM sodium pyruvate and 1× penicillin/streptomycin.

Standard whole-genome sequencing (WGS) and Replication timing whole-genome sequencing (Repli-seq) were applied to CRUK0557-CL and CRUK0977-CL in this study. For standard WGS, genomic DNA (gDNA) was extracted from cultured cells after 13 (CRUK0557-CL) or 8 (CRUK0977-CL) passages using the Qiagen AllPrep kit according to the manufacturer’s instructions. The quantity and quality of the gDNA were assessed using Invitrogen’s Qubit 1x double-stranded DNA (dsDNA) high sensitivity (HS) assay kit on the Qubit Flex Fluorometer (Thermo Fisher Scientific, Inc.) and Agilent Technologies’ gDNA ScreenTape on the TapeStation 4200 (Agilent Technologies, Inc.), respectively. 400 ng of each gDNA was transferred to a microTUBE plate and underwent mechanical shearing using the LE220-plus focused-ultrasonicator (Covaris) followed by a clean-up using SPRI select beads (Beckman Coulter, Inc.). Libraries were prepared with the NEBNext® Ultra II DNA library prep kit for Illumina (New England Biolabs (NEB) #E7645S) using an input of 250 ng of sheared DNA. Unique dual index primers from NEBNext® Multiplex Oligos for Illumina were used at a concentration of 15 μM (NEB #E6440S). Four cycles of PCR amplification were performed. SPRI select beads were used for clean-ups and size selection (Beckman Coulter, Inc.). 150 bp paired-end sequencing was performed at the Francis Crick Institute using the Illumina NovaSeq 6000 to achieve 30x coverage (Illumina, Inc.).

The whole-genome sequencing data was processed using the nf-core Sarek pipeline^70^. Briefly, FASTQ files were trimmed using TrimGalore (v0.6.4) and were aligned to hg38 using bwa-mem (v07.17). Duplicates were marked and BQSR was done using GATK4 (v4.1.7). Variant calling was performed using Strelka (v2.9.10) with default parameters. In addition, variants with less than 5 alternative reads and a total depth of less than 30 in the tumour as well as 1 or more alternative reads in the germline were filtered out. Purity, ploidy and copy number calling were performed using AscatNGS (v4.2).

### Repli-seq protocol

#### BrdU-labelling and sorting cells

The protocol used in this study was modified from previously published studies (Supplementary Fig. 1)^71,72^. Asynchronized cells were grown in flasks for at least 48 h to achieve at least 10^7^ cells with confluency of less than 80% and were labelled with 50 μM bromodeoxyuridine 5-bromo-2′-deoxyuridine (BrdU; 100 µl BrdU at 1.5 mg/ml were added per 10 ml of culture media to achieve a final concentration at 50 µM) for 2 h at 37 °C in a CO_2_ incubator in the dark. Cells were then harvested and washed using ice-cold PBS twice after spinning down (340 × *g* for 5 min at 4 °C in the dark). Cell pellets were fixed in a mixture of 7.5 ml ice-cold 100% ethanol and 2.5 ml PBS containing 2% (v/v) FBS. The fixed cell pellets were incubated on ice for at least 30 min or stored at −20 °C.

After washing twice using ice-cold washing buffer (PBS with 1% FBS), DNA contents in the fixed cell pellets were washed twice using ice-cold washing buffer and stained using 200 μl propidium iodide (PI) buffer per million cells which contained 50 μl 50 μg/ml ribonuclease A (Sigma; P4170) and 150 μl 100 μg/ml PI (Sigma; R5125). Prior to sorting cells using fluorescence-activated cell sorting (FACS), to get a single-cell suspension, the cell pellets were disaggregated by passing through a 25G needle using a 1 ml syringe and filtered through a 40 μm nylon mesh to remove any clumps or aggregates.

During FACS, BrdU-labelled and PI-stained cells for each cell line were sorted into 3 fractions based on the DNA contents (G1, Early S, Late S) using a FACS Aria II cell sorter. In brief, the gating strategy during FACS was as follows. Cells were initially gated using forward scatter (FSC) versus side scatter (SSC) to exclude debris and aggregates. Subsequently, sequential gating strategies were employed to identify single cells, including gating based on FSC-A versus FSC-H, SSC-A versus SSC-W, and propidium iodide (PI)-A versus PI-W to exclude apoptotic cells and doublets further. The G0/G1 and G2/M phases were distinguished by the peak of PI staining intensity, incorporating DNA content with cell size (FSC-A) as a reference. To ensure accuracy, early S and late S phases were equally gated between the G0/G1 and G2/M phases, while the edges of each population were avoided to minimise potential artefacts.

After cell sorting, cell pellets were then digested for DNA extraction and purification using a phenol-chloroform extraction protocol as previously reported^73^.

1. Add 1 volume of phenol: chloroform: isoamyl alcohol (25:24:1) to each sample, and vortex or shake by hand thoroughly for approximately 20 s;
2. Centrifuge at room temperature for 5 min at 16,000 × *g*;
3. Carefully remove the upper aqueous phase, and transfer the layer to a fresh tube (Be sure not to carry over any phenol during pipetting);
4. Add 4 µl glycogen and then 1 volume of propanol, mix well. Store at −80 °C around dry ice for >1 h;
5. Centrifuge at 16,000 × *g* for 30 min at 4 °C. Discard the supernatant, add 750 µl of cold 70% ethanol to the pellet;
6. Centrifuge at 16,000 × *g* for 5 min at 4 °C. Remove all ethanol (using 10 µl tips) as much as possible, let the pellet air dry;
7. Resuspend the pellet in 50 µl of 1× low TE (10 mM of 1 M Tris-HCl and 0.01 mM of 0.5 M EDTA) at 37 °C for 1 h with 350–400 rpm shaking.

Then the purified DNA samples were stored at 4 °C in the dark.

#### Library construction

Purified DNA was then fragmented using a Covaris ultrasonicator to achieve an average length of 200 bp. The NEBNext Ultra DNA Library Prep Kit for Illumina (NEB; E7370) and the NEBNext Multiplex Oligos for the Illumina kit were applied to construct the library by ligating adaptors before BrdU immunoprecipitation. Two commercially available kits were used at this step, by following the manufacturer’s instructions: the NEBNext Ultra DNA Library Prep Kit which was used for end repair, and the NEBNext Multiplex Oligos for Illumina kit which was used for adaptor ligation and some enzyme treatment. Firstly, the end repair enzyme and the reaction buffer were added to the fragmented DNA. After a thorough mixing and quick spinning down, the sample was put in the thermocycler starting from 20 °C for 30 min, followed by 65 °C for another 30 min. After the end repair, the sample could be held at 4 °C before the next step. Second, according to the manufacturer’s instructions for the NEBNext Multiplex Oligos for Illumina kit, the NEBNext Adaptor for Illumina, the Ligation Enhancer and other buffer included in the kit were added to the repaired samples from the last step, followed by the incubation at 20 °C for 15 min. Lastly, the uracil-specific excision reagent (USER) enzyme digestion from the NEBNext Multiplex Oligos for Illumina kit was performed by adding the USER enzyme to the ligated sample for further incubation at 37 °C for 15 min. The digested DNA sample was then purified using the QIAquick PCR Purification Kit and the DNA was eluted in 50 μl molecular biology grade water.

#### BrdU immunoprecipitation

Eluted DNA samples after the library construction were immunoprecipitated with 40ul mouse anti-BrdU antibody at 12.5 μg/ml (Monoclonal, Clone B44. BD Biosciences; 347580) for 20 min at room temperature with constant rocking and then followed by 20 μg goat anti-mouse secondary antibody at a concentration of 1 mg/ml (IgG-Alexa Fluor 488. Abcam; ab150129). The procedures in detail for BrdU immunoprecipitation were slightly modified from literature^72^ as follows.

First, 450 μl of TE buffer (10 mM Tris-HCl and 1 mM EDTA in ddH_2_O, stored at room temperature) was added to each DNA sample which was eluted in 50 μl of H_2_O. Next, the DNA sample was denatured at 95 °C for 5 min and then cooled down on ice for at least 2 min. Another 60 μl 10× IP buffer (to prepare 50 ml 10× IP buffer stored at room temperature: 28.5 ml of ddH_2_O, 5 ml of 1 M sodium phosphate, 14 ml of 5 M NaCl, 2.5 ml of 10% (wt/vol) Triton X-100) and 40 μl of 12.5 μg/ml mouse anti-BrdU antibody (to achieve a final concentration of 0.83 μg/ml) were added to the denatured DNA in each tube, followed by the incubation for 20 min at room temperature with constant rocking. Next, 20 μg of the secondary antibody, the goat anti-mouse IgG (IgG-Alexa Fluor 488. Abcam; ab150129) at a concentration of 1 mg/ml at stock, was added to each tube to achieve a final concentration of 0.03 μg/μl. The incubation was extended to overnight at 4 °C to improve the efficiency of immunoprecipitation.

Next, the supernatant in the sample tube was removed after centrifuging at 16,000 × *g* for 5 min at 4 °C. Then, 10ul tips were used to remove as much remnant supernatant as possible after spinning it down quickly. The pellet was washed by 750 μl ice-cold 1× IP buffer twice, and then re-suspended in 200 μl of digestion buffer (to prepare 50 ml digestion buffer stored at room temperature: 50 mM Tris-HCl, 10 mM EDTA and 0.5% (wt/vol) SDS in ddH2O) and freshly added 0.25 mg/ml proteinase K for further incubation overnight at 37 °C with 300 rpm shaking. At the end of incubation, another 1.25 μl of 20 mg/ml proteinase K was freshly added to each tube which was then incubated for another 1 h at 56 °C. DNA was then purified using the QIAquick PCR Purification Kit following the manufacturers’ instructions and then eluted in 20 μl low TE buffer (10 mM Tris-HCI, 0.1 mM EDTA in molecular biology grade water).

#### Validation of replication timing

Before moving to the next step, we performed qPCR (quantitative polymerase chain reaction) of DNA samples from G1, early and late S phases to validate whether this protocol worked to call replication timing properly. Primers of three known early (*HBA1*, *MMP15*, *BMP1*) and four late (*PTGS2*, *SLITRK6*, *ZPF42* and *DPPA2*) replicated genes were used as previously reported^71^. Meanwhile, mitochondrial DNA sequences were used as an internal control, as mitochondrial DNA is supposed to be equally represented in early and late S phase fractions.

The primers for the seven genes used for primary validation of replication timing:

*HBA1:*

- Forward, GACCCTCTTCTCTGCACAGCTC
- Reverse, GCTACCGAGGCTCCAGCTTAAC

*MMP15:*

- Forward, CAGGCCTCTGGTCTCTGTCATT
- Reverse, AGAGCTGAGAAACCACCACCAG

*BMP1:*

- Forward, GATGAAGCCTCGACCCCTAGAT
- Reverse, ACCCGTCAGAGACGAACTTGAG

*PTGS2:*

- Forward, GTTCTAGGCTGGTGTCCCATTG
- Reverse, CTTTCTGTACTGCGGGTGGAAC

*NETO1:*

- Forward, GGAGGTGGAATGCTAGGGACTT
- Reverse, GCTGAGTGTGGCCTTAAGAGGA

*SLITRK6:*

- Forward, GGAGAACATGCCTCCACAGTCT
- Reverse, GTCCTGGAAGTTGAGTGGATGG

*ZFP42:*

- Forward, CTTGTGGGGACACCCAGATAAG
- Reverse, AACCACCTCCAGGCAGTAGTGA

*DPPA2:*

- Forward, AGGTGGACAGCGAAGACAGAAC
- Reverse, GGCCATCAGCAGTGTCCTAAAC

The primers for mitochondrial DNA are as follows:

- Forward, CTAAATAGCCCACACGTTCCC
- Reverse, AGAGCTCCCGTGAGTGGTTA

To quantify the replication timing using qPCR, we calculated the relative abundance of G1, early and late S samples per gene per cell line using this equation:

1 $$\begin{array}{c}{RelativeAbundace}\left({s}_{i},{{gene}}_{j}\right)=\frac{{2}^{\left({{Ct}}_{{{gene}}_{j}}\left({S}_{i}\right)-{{Ct}}_{{mitochondria}}\left({s}_{i}\right)\right)}}{{\sum }_{t=1}^{3}{2}^{\left({{Ct}}_{{{gene}}_{j}}\left({S}_{t}\right)-{{Ct}}_{{mitochondria}}\left({s}_{t}\right)\right)}}\\ {with}\, {i}=1,2,3\, {and}\, {j}=1,...,n\end{array}$$

In this equation, ${S}_{i}$ represents one of the three FACS sorted samples (${S}_{i}\epsilon \{G1,\,{earlyS},{lateS}\}$) and ${{gene}}_{j}$ the *j-th* gene of *n* total genes. Therefore, ${{Ct}}_{{{gene}}_{j}}({S}_{i})$ describes the Ct value of the *i-th* FACS sorted sample and the *j-th* gene. Similarly, ${{Ct}}_{{mitochondria}}({s}_{i})$ describes the Ct value of the mitochondrial DNA abundance of the *i-th* sample. The ${RelativeAbundace}({s}_{i},{{gene}}_{j})$ was calculated for each cell line separately.

#### Multiplex WGS

Purified BrdU-immunoprecipitated DNA samples were applied for indexing and PCR amplification using the NEBNext Ultra II Kit (NEB; M0544). Primers annealing to the adaptors to determine the optimal PCR cycle number were as follows:

- adqPCR_Forward: ACACTCTTTCCCTACACGACGC
- adqPCR_Reverse: GACTGGAGTTCAGACGTGTGC

Next, PCR reactions were purified using AMPure XP beads (Beckman Coulter; A63880) and DNA was eluted in 10 mM Tris-HCl. After quantifying the DNA concentration of each sample using the Qubit dsDNA HS Assay Kit (Life Technologies; Q32854), libraries were pooled, followed by checking the size distribution of DNA fragments. Whole genome sequencing with 100 bp paired end reads was performed on an Illumina HiSeq4000 with 6 or 12 samples per lane.

### Repli-seq bioinformatics pipeline

The bioinformatics pipeline was based on the pipeline provided by the 4D Nucleosome Data Coordination and Integration Center^74^ in combination with the pipeline published in Marchal et al.^72^.

#### Alignment

To clean up the raw sequencing data and to remove any unwanted left-over adaptor sequences, TrimGalore (v0.6.5) a wrapper tool around Cutadapt^75^ and FastQC (https://www.bioinformatics.babraham.ac.uk/projects/fastqc/), was applied with default settings to perform quality and adaptor trimming for each set of paired-end fastq files. The resulting fastq files were provided to bwa-mem (v0.7.17)^76^ for alignment to both reference genomes hg19 and hg38 in two separate runs to account for differences in the genome build used in the downloaded WGS datasets of lung and breast tumours. The samtools software (v1.8)^77^ was used for further quality and filtering steps. In cases where one Repli-seq run resulted in multiple sets of fastq-files, samtools merge (flags: -n -f -b) was used to combine them into one. Afterwards samtools view (flags: -bhq 20) and samtools sort (flags: -m 16G –threads 4) were applied to exclude reads with a mapping quality lower than 20 and to sort reads in the bam files regarding their genomic position, respectively. Samtools stats were applied to quality check the different alignment and filtering steps. To remove duplicated reads, samtools rmdup was applied resulting in the final bam files which were indexed by samtools index.

#### Calculation of the replication timing signal

To estimate the replication timing (RT) signal measured as the log2-transformed ratio of early to late replicated reads, the genome was split into 1 kb and 50 kb non-overlapping windows using bedtools makewindows (v2.26)^60^. Next, the RPKM metric was calculated per bin to normalise the read counts obtained by bedtools coverage (flags: -counts -sorted) for sequencing depth and window size for early and late replicated reads separately. Windows with a total RPKM value across the two cell cycle states, early S and late S, less than 0.1 and windows with an RPKM value equals 0 for early and late replicated reads were excluded from the analysis due to low coverage regions. The RT signal was calculated as the log2-transformed ratio of early versus late replicated RPKM values per bin using bash commands. The following steps were performed in the statistical environment R (v3.5.1) and restricted to chromosomes 1–22. To make the RT signal distribution of different cell lines comparable to each other, the RT signals were quantile normalised relative to the RT signal of the T2P cell line using the normalize.quantiles.use.target method provided by the R-package preprocessCore (v1.44). For noise reduction purposes, loess smoothing with a span of 300 kb was applied per chromosome using the loess basic R function. A minority of bins presented a big difference in their RT values before and after smoothing which was attributed to neighbouring bins with NA values. NAs were introduced in regions that were previously filtered out due to low mapping quality or low coverage. To prevent potential biases caused by smoothing artefacts, bins with an absolute difference greater than 3 between their RT values before and after smoothing were excluded for further analysis. The smoothed values were used as the final replication timing signal with positive values representing early replicated regions and negative values representing late replicated regions.

#### Analysis of the effect of copy number alterations on the replication timing signal

To assess the effect of copy number alterations on the replication timing analysis, available copy number data have been obtained from COSMIC and analysed for a subset of lung adenocarcinoma (A549, H1650, H1792, H2009) and breast cancer (MCF-7, MDA453, T47D) cell lines. Specifically, the PICNIC^61^ algorithm has been previously used to infer these copy number data. Copy number alterations were classified relative to the ploidy of the cell lines into three distinct groups: losses, neutral, and gains. To do this, the copy number of every 50 kb genomic bin used for the replication timing analysis was determined by using the total copy number of the segment that covered most of the bin. In very few cases (less than 200), where no segment was overlapping, the copy number of the closest segment was used. As such, the rounded integer value of tumour ploidy has been used as a reference to identify copy number alterations and every genomic bin was classified as gained if the assigned copy number was greater than the ploidy and as lost if the copy number was less than the ploidy. To adopt a conservative approach as in previous studies^78^, the integer rounding of ploidy that results in the least number of copy number events was chosen for the final results. Lastly, the T signal of genomic regions with different copy number classifications was compared. Since the number of genomic regions was substantially different across different classes, we applied a bootstrapping approach to assess if the median of the different classes of replication timing values was different. To do this, 10,000 gained, lost and neutral bins were randomly sampled, and the median replication timing signal was calculated per copy number status. This step was repeated 10,000 times and the results are displayed in Supplementary Fig. 2D.

#### Pipeline validation with Repliscan as an orthogonal method

Repliscan^79^, a published tool to classify replication timing regions across the genome, was used as orthogonal validation for the replication timing signal estimated as the log2-transformed ratio between early and late replicated reads. The resulting bam files from the alignment step were used as input. To make the results as comparable as possible similar parameters for the calculation of the log2-ratio-based RT signal were used, including the same window sizes of 1 kb and 50 kb. To account for differences in the DNA composition across the genome, Repliscan normalises for sequencing ability using non-replicating G1 DNA or a combination of early S phase and late S phase replicating DNA for correction. Given that not all cell lines obtained from ENCODE provided data for G1 cells and to stay consistent across all cell lines, the option of combining early and late replicated reads for copy number correction was used. Repliscan provides a replication timing signal for early and late replicated reads separately and classifies genomic regions as either early (ES), late (LS) or early and late (ESLS) replicated. The comparison of the timing classifications of the 1 kb and 50 kb bins across the genome provided by the log2-ratio-based RT signal and Repliscan yielded a high concordance of more than 90% overlap between the two methods in all cell lines. The majority of discordant bins consisted of cases where the replication timing was not confidently detectable in one of the methods rather than reporting opposite timings.

#### Validation of the Repli-seq protocol and the bioinformatics pipeline using biological replicates

To validate the reproducibility of the Repli-seq protocol and the bioinformatics processing pipeline, biological replicates of the T2P and H1650 cell line were processed and the replication timing signal at different stages of the pipeline was compared between a pair of replicates, for both 1 kb and 50 kb windows. In addition, our in-house Repli-seq protocol was applied to the A549 cell line which is also available on ENCODE. Both sets of fastq files derived from different Repli-seq protocols were processed through the bioinformatics pipeline and the resulting replication timing signals were compared between each other for 1 kb and 50 kb windows (Supplementary Fig. 2A–C). The Pearson correlation test was calculated in R (v3.5.1) for each pair of replicates at different normalisation stages of the pipeline. This analysis resulted in high agreement between the replicates which ensures great robustness and reproducibility of our experiments. The additional step of comparing results for the A549 cell line derived from our IN-STUDY protocol and the protocol used for publicly available data on ENCODE issued high similarity confirming the combined use of both datasets. For downstream analyses, replicates of the T2P and H1650 cell lines were combined by calculating the average replication timing signal for each 1 kb and 50 kb bin whereas for A549 the results of our IN-STUDY sequenced A549 cell line were used alone.

### Identification of altered replication timing (ART) regions

To identify 50 kb bins that present significantly different RT signals between normal and cancer, the RT signal between replicates of the T2P, H1650 and A549 cell lines were compared to estimate differences due to random background noise induced by technical biases. The thresholds for altered replication timing identification were chosen based on the 0.5%-quantile (−1.66) and the 99.5%-quantile (1.45) of the combined distribution of differences in RT values between the replicates (Supplementary Fig. 4). This means <1% of values were assumed to be artifactual or outliers. Given that the distribution of differences is centred around 0, a symmetrical threshold of |2| was used to classify genomic regions with replication timing alterations. Genomic bins that presented absolute differences in the RT signal between cancer and tissue-of-origin greater than |2| but exhibiting the same RT classification (early or late in both cancer and normal) were excluded from further analyses due to uncertainties whether these regions were altered or not.

#### Replication timing domains

Given that neighbouring 50 kb bins with the same replication timing are likely part of the same replication timing domain means that they are not independent. Therefore, adjacent bins with the same type of altered replication timing were likely caused by the same alteration event of a certain replication timing domain. For this reason, for some analyses adjacent bins with the same RT or ART classification were combined into one RT or ART domain.

#### Identification of ART genes

In order to identify genes with an altered replication timing between tissue-of-origin and cancer we calculated the mean RT signal using the 1 kb window results, for each gene and applied the same thresholds as described above.

#### Recurrent and shared ART regions

Recurrent ART regions were classified as genomic bins that were identified as presenting ART in all cancer cell lines relative to the tissue-of-origin within a given cancer type. Shared ART regions were defined as genomic bins that were altered in at least 2 cancer cell lines but not all within a given cancer type. ART regions that were only identified in one of the cell lines were classified as being uniquely altered. ART regions that were present in at least 2 cell lines or all (shared and recurrent) were used for most of the analyses in this study. In order to test if the observed overlaps were significantly higher than random, a bootstrapping approach was applied to randomly distribute ART regions, summarised as replication timing domains, across the genome for each cell line. Only regions of the genome that were early replicated in the tissue-of-origin have the potential to become Early_N_-to-Late_T_ altered replicated in cancer and vice versa for Late_N_-to-Early_T_ replicated. Therefore, the sampling of Early_N_-to-Late_T_ domains was restricted to regions that were early replicated in the tissue-of-origin and vice versa for Late_N_-to-Early_T_ domains. The altered replication timing domains were randomly distributed across the genome 1000 times and the number of overlapping ART bins between cell lines of the same cancer type were counted during each iteration. The 95% confidence interval of the bootstrapped distribution of overlapping bins was used as background distribution to test the observed fractions for significance.

### Integration of DNA and RNA sequencing data from lung and breast cancer tumours

#### Copy number adjusted mutation load

Differences in the copy number of the DNA across the genome can influence the accumulation and detection of mutations. More genomic material might increase the likelihood of accumulating mutations and lead to higher coverage for sequencing leading to a higher detection rate of mutations. To account for these differences, mutations of the lung and breast cancer tumours were not only counted in 50 kb bins but corrected for differences in copy number. For this, the minor (nMinor) and major (nMajor) copy number estimate of the genomic segment that a certain mutation was located on was assigned to each mutation. This information in combination with the mutation’s variant allele frequency (VAF), the tumour’s purity and the total copy number in the germline (normalCN) was used to estimate the number of copies that presented the mutation (mutCN):

2 $${mutCN}=\frac{{VAF}}{{purity}} * \left({purity} * ({nMinor}+{nMajor})+\frac{{normalCN}}{1-{purity}}\right)$$

Instead of counting each mutation as 1 in the 50 kb bins, each mutation was counted as their mutCN divided by the total copy number (nMinor + nMajor) of the segment that this mutation was located on.

3 $${{mutLoad}}_{j}=\sum\limits_{i=1}^{{n}_{j}}\frac{{{mutCN}}_{i}}{({{nMinor}}_{i}+{{nMajor}}_{i})}$$

with ${n}_{j}$ representing the total number of mutations and ${{mutLoad}}_{j}$ the final copy number corrected mutation load of the *j-th* 50 kb bin (Fig. 1C). The mutation load in 50 kb bins was calculated on a cohort level and a per-tumour level. For the cohort analysis, all mutations within a certain 50 kb bin were pooled together across all tumours of a certain cohort for the mutation load calculation.

#### ART timing relative to the mutation accumulation in the most recent common ancestor (MRCA)

For each mutation, its CCF and a 95% confidence interval (CI) (as described in ref. ^80^) were calculated. Mutations with an upper 95%-CI greater equals 1 were classified as clonal and everything else as subclonal. The copy number adjusted mutation load was calculated for the aggregated data of the BRCA and LUAD cohort by only considering clonal mutations. The resulting mutation load estimates were z-transformed to be able to compare the final results across the two cancer types. Next, the mean mutation load for the different altered and unaltered replication timing regions was estimated by applying a bootstrapping method to account for the high variability in the number of 50 kb bins classified as presenting different types of altered or unaltered RT. The lowest number of bins regarding the different timing classifications was determined per cancer type (BRCA: 4128, LUAD: 1498) and used as the number of bins that were randomly sampled with replacement from each timing classification. The sampling step was conducted 10,000 times and during each iteration, the mean mutation load per RT and ART classification was calculated. This resulted in a distribution of mean mutation load estimates for each timing per cancer type. To estimate the proportion of mutations that were likely accumulated before the Late_N_-to-Early_T_ alterations during tumour evolution, the absolute difference between the mean mutation load estimates in Late_N_-to-Early_T_ minus unaltered Early_N+T_ replicated regions was calculated per iteration. This difference was normalised by the absolute difference in mutation load between unaltered Late_N+T_ minus Early_N+T_.

4 $${d\left({Late}-{to}-{Early}\right)}_{i}=\frac{{{|}{mutLoad}({Late}-{to}-{Early})}_{i}{-}{mutLoad}{({Early})}_{i}{|}}{{|}{mutLoad}({Late})_{i}{{-}}{mutLoad}{({Early})}_{i}{|}}$$

5 $${d\left({Early}-{to}-{Late}\right)}_{i}=	\frac{{{|}{mutLoad}({Early}-{to}-{Late})}_{i}-{mutLoad}({Late})_{i}{|}}{{|}{mutLoad}({Late})_{i}-{mutLoad}({Early})_{i}{|}} \\ {with} \ i=	1,...,10000$$

The equivalent was calculated to estimate the proportions of mutations accumulated before the Early_N_-to-Late_T_ alterations occurred during tumour evolution. This resulted in a distribution of proportions accumulated prior to ART (Supplementary Fig. 8F). The mean values of these proportions were used separately as the final timing estimates for the two different RT shifts relative to mutation accumulation in the most recent common ancestor. A low proportion suggests ART to be an early evolutionary event whereas a high proportion means that the ART event occurred closer to the emergence of the MRCA.

#### Simulations of different ART time points

To validate the estimation of the ART timing relative to the mutation accumulation in the MRCA, different fractions of mutations that were accumulated before the shift in RT were simulated as different time points. For this, a fixed mutation rate in early and late replicated regions was assumed, which was estimated based on the fractions of mutations per Mb in unaltered RT regions for each cancer type (Supplementary Fig. 8A). A higher disparity in the fraction of mutations accumulated in Late_N+T_ versus Early_N+T_ replicated regions was observed in lung cancer in comparison to breast cancer. For this reason, in breast cancer, the mutation rate in late replicated regions was set to be 1.3 times the mutation rate in early replicated regions whereas in lung cancer the mutation rate was set to be 1.5 times higher in late versus early replicated regions for the simulations. Next, the accumulation of mutations during 10,000 iterations (representing cell divisions) in 1000 genomic bins with different replication timings and corresponding mutation rates was simulated to explore the resulting mean mutation load patterns when certain proportions of mutations were accumulated before the ART event (Supplementary Fig. 8B). Two-thirds of the 1000 bins were initiated as late replicated (660 bins) and one third as early replicated (340 bins) which was based on the observed fractions of the genome harbouring early versus late replicated regions in all cell lines (Supplementary Fig. 3). The mutation rate in early replicated bins was set to accumulate a mutation with a likelihood of 0.1 in each iteration whereas late replicated regions accumulated a mutation with a likelihood of 0.13 in breast cancer and 0.15 in lung cancer. If a bin was selected to present ART, the mutation rate changed accordingly for the remaining iterations. Given that approximately 15% of the genome displayed ART in BRCA and 8% in LUAD (Fig. 2B) similar fractions of the 1000 simulated bins were chosen to switch their RT at different time points during the 10,000 iterations, respectively. In both cancer types, half of the replication timing alterations presented a switch from Early_N_-to-Late_T_ whereas the other half presented a change from Late_N_-to-Early_T_, which was considered in the simulations. The different time points for ART were simulated as different proportions of iterations, ranging from 0 to 1 in 0.01 steps, that accumulated mutations prior to ART. This means after a certain iteration, the replication timing and mutation rate were changed for a subset of bins and the remaining iterations accumulated mutations regarding the updated mutation rates. The mutations accumulated throughout the 10,000 iterations were counted for each of the 1000 bins illustrating the mutation load in bins across the genome. Afterwards, the bootstrapped mean mutation load values and the differences $d({Early}-{to}-{Late})$ and $d({Late}-{to}-{Early})$ were calculated as described above in Eqs. (4) and (5) (Supplementary Fig. 8C–E). Comparing the simulated differences to the observed differences revealed that the estimated proportions of mutations accumulated prior to ART represent an appropriate timing estimate when assuming constant mutation rather than throughout tumour development (Supplementary Fig. 8F).

#### Per-tumour analysis of ART timing relative to the mutation accumulation in the most recent common ancestor (MRCA)

The copy number adjusted mutation load was calculated for each BRCA and LUAD tumour separately by only considering clonal mutations. The resulting mutation load estimates were z-transformed and the mean mutation load for the different altered and unaltered RT regions was estimated by applying the same bootstrapping method as used for the aggregated cohort analysis. This resulted in a distribution of mean mutation load estimates for each timing per tumour. To be able to detect a shift in the mutation distribution in ART versus unaltered RT regions a significant difference in the mutation load between unaltered Early_N+T_ and Late_N+T_ replicated regions was required. Therefore, for each tumour a bootstrapping *p*-value was calculated as the number of iterations where the mean mutation load value in Early_N+T_ replicated regions was greater than or equal to the mutation load value in Late_N+T_ replicated regions divided by the total number of iterations (10,000). Only 3 BRCA tumours presented a bootstrapping *p*-value < 0.001 and therefore a significant shift in the mutation load between unaltered Early_N+T_ and Late_N+T_ replicated regions, hence BRCA was excluded from the per-tumour ART timing analysis. A bootstrapping *p*-value < 0.001 was detected for 178 LUAD tumours for which ART timing was estimated on a per-tumour level in the same way as for the cohort analysis.

#### Paired RT and mutation analysis in PDCs derived from two TRACERx LUAD tumours

The T2P cell line was used as a normal reference to identify ART regions in the two PDCs derived from two patients with LUAD in the TRACERx study (CRUK0557-CL and CRUK0977-CL). The copy number adjusted mutation load was calculated separately for each PDC by only considering clonal mutations. The resulting mutation load estimates were z-transformed and the mean mutation load for the different altered and unaltered replication timing regions within the corresponding cell line was estimated by applying the same bootstrapping method as used for the cohort and per-tumour analysis. This resulted in a distribution of mean mutation load estimates for both PDCs, where the mutation and RT information were derived from the same sample.

#### Identification of ACC regions and their correlation with mutation accumulation across the genome

Only Hi-C data for the normal breast reference cell line HMEC was available and not for the normal lung cell line T2P, hence altered chromatin compartment (ACC) regions were only identified for the two BRCA cell lines MCF-7 and T47D and no LUAD cell line. For this the in situ Hi-C values in 50 kb bins from T47D were compared against the in situ Hi-C values from HMEC whereas the intact Hi-C HMEC values were compared against the intact Hi-C values from MCF-7. Genomic bins that presented an absolute difference in Hi-C values between tissue-of-origin and cancer greater than |0.03| and a shift from A compartment in normal to B compartment in cancer were classified as A_N_-to-B_T_ altered regions. Similarly, genomic bins that presented an absolute difference in Hi-C values greater than |0.03| and a shift from B compartment in normal to A compartment in cancer were classified as B_N_-to-A_T_ altered regions. Genomic bins that presented an absolute difference in Hi-C values between cancer and tissue-of-origin greater than |0.03| but exhibited the same chromatin compartment classification (A or B in both cancer and normal) were excluded from further analyses due to uncertainties whether these regions were altered or not. Unaltered A compartment regions are referred to as A_N+T_ and unaltered B regions as B_N+T_. The same methods as for the estimation of ART timing relative to mutation accumulation in the MRCA were applied for the investigation of the association of ACC with the accumulation of mutations across the genome with the only difference being that shared ART regions were used previously and here the analysis was conducted for ACC regions in MCF-7 and T47D separately. The copy number adjusted mutation load of clonal mutations for the BRCA cohort was analysed and z-transformed. The mean mutation load for the different altered and unaltered chromatin compartment regions was estimated by applying the same bootstrapping method as used for the ART analysis.

#### Comparison analyses of the association of alterations in RT and chromatin structure with the mutation distribution across the genome

To explore whether the variability in local mutation burden across the genome in BRCA tumours can be better explained by RT or chromatin structure in normal or cancer cells, we conducted multiple linear regression models. First, we applied 4 univariate linear regression models for both BRCA cell lines (MCF-7 and T47D) separately with the mutation load in BRCA tumours as an independent variable and the RT signal (Repli-seq log2-ratio) and chromatin compartment signal (Hi-C values) in normal (HMEC) and cancer cells as the dependent variable, respectively. Afterwards, we compared the resulting R^2^-values between the 4 models for both BRCA cell lines. To evaluate whether RT in cancer is still the best predictor in a multivariate setting, we z-transformed the RT signal and chromatin compartment signal in normal (HMEC) and cancer (MCF-7 and T47D) cells and used the resulting values as dependent variables in a multivariate linear regression model with mutation load in BRCA tumours as independent variable. Due to the z-transformation, we were able to compare the absolute estimate values between the different values to explore which of them contributes the most in explaining the variability in the mutation distribution across the genome.

#### De novo extraction of replication timing specific mutational signatures

A Hierarchical Dirichlet Process (HDP) Model^35^ implemented in the hdp R-package (v0.1.5) available on GitHub (https://github.com/nicolaroberts/hdp) was applied to extract de novo signatures for the different altered and unaltered RT region for BRCA and LUAD separately. For this, the trinucleotide profile of mutations located in the different altered and unaltered RT regions was constructed per patient and used as input. Using an HDP model to infer mutational signatures enabled the definition of hierarchies of relatedness between samples via the tree of parent Dirichlet Process (DP) nodes. This provided the opportunity to derive mutational signatures in different replication timing regions per tumour without neglecting tumour-specific signatures. The HDP was structured to have one grandparent DP, four parent DPs representing the different replication timing categories and the number of tumours within a certain cohort as child DPs (BRCA = 482 and LUAD = 470) per parent with the trinucleotide context of the different tumours for the corresponding replication timing region assigned. If a tumour harboured less than 50 mutations for one of the replication timing categories, it was excluded from the corresponding parent for this analysis. Signatures that were identified to be commonly active in lung and breast cancer were included as priors accordingly (BRCA: SBS1, SBS2, SBS3, SBS5, SBS6, SBS8, SBS13; LUAD: SBS1, SBS2, SBS4, SBS5, SBS13, SBS40). This means for each of these signatures a cluster was initialised at the start of the algorithm and their trinucleotide pattern was provided as prior knowledge to force the algorithm to look for these signatures in the data. 10 random clusters were initialised in addition to detecting de novo signatures that were not included in the list of priors. The model was initialised by applying the function hdp_init(). The trinucleotide profiles were assigned to the leaves by hdp_setdata() and the nodes were activated by dp_activate(). By applying hdp_posterior() 15 times with different seeds 15 independent posterior sampling chains were constructed followed by 10,000 burn-in iterations and the collection of 100 posterior samples off each chain with 200 iterations between each. The hdp_multi_chain() function was applied to combine the results of the 15 chains from which the final components were extracted using hdp_extract_components(). These components were compared to the signatures reported in Degasperi et al.^38^ combined with the signatures reported on COSMIC (v3.2). For this, the cosine similarity between the hdp-derived components and the signatures provided by the public datasets was calculated by the function cosine() of the lsa R-package (v0.73.2). If a component displayed a cosine similarity greater than 0.85 with any of the known signatures, the corresponding signatures were assigned to that component. Some signatures were found to be often co-occurring in cancer, such as SBS1 and SBS5, which makes it challenging to identify them separately during de novo signature extraction. In these cases, the Expectation Maximisation (EM) algorithm was used to identify pairs of signatures that might explain the observed signature. The identified pair was then used to reconstruct the observed signature considering the weights provided by the EM algorithm. If the reconstructed signature presented a cosine similarity greater than 0.85 with the observed signature, the signature was recognised as a combination of the identified pair. In that instance, the exposure of the observed signature was split based on the weights provided by the EM algorithm for further analyses. The full HDP pipeline for de novo signature extraction can be accessed from https://github.com/McGranahanLab/HDP_sigExtraction.

#### Identification and investigation of APOBEC3-mediated omikli events

The hyperClust algorithm (https://github.com/davidmasp/hyperclust) provided in the form of a nextflow pipeline by Mas-Ponte and Supek^42^ was applied to identify kataegis and omikli events across the genome for BRCA and LUAD tumours. Nextflow version 0.30.0 was used to run the pipeline with all default parameters as described on GitHub. APOBEC mutations were classified based on their stringent mutation pattern of C > T and C > G mutations in a TCW motif with the C in the middle representing the mutated cytosine and W corresponding to either A or T^41^. Mutations that showed a C or G in the position of the W were excluded from this analysis, due to the uncertainty of whether these mutations were mediated by APOBEC or not. Fisher’s tests were applied to statistically test for significant enrichment of APOBEC-mediated omikli mutations in cancer genes related to their RT or ART. For this, the number of coding APOBEC and non-APOBEC mutations in an omikli and unclustered manner within cancer-associated genes were counted for the different replication timing regions. The odds ratio of omikli and unclustered mutations in an APOBEC context versus without was tested for significance for the different replication timing regions separately.

#### Differential expression analysis

To identify differentially expressed genes (DEGs), RNA-seq data from tumour and normal tissues within the same patient enrolled in TCGA were analysed using the R-package, DESeq2^81^. In this analysis, the log2-transformed fold change (log2FC) of the gene expression in tumour versus normal tissue was calculated for every gene. A gene was classified as being significantly up-regulated in cancer if the resulting *p*-value < 0.05 and the log2FC > 1. Similarly, a gene was classified as being significantly down-regulated in cancer if the resulting *p*-value < 0.05 and the log2FC < 1. Genes that did not meet any of these criteria were classified as not differentially expressed in cancer compared to normal tissues.

#### Analyses of gene expression and copy number alterations in genes with and without ART

To investigate whether genes with ART presented a significant change in their expression in tumour versus normal than unaltered RT genes, a bootstrapping method was applied to estimate the log2FC distribution of early and late replicated genes in the tissue-of-origin. For this, only genes that presented at least 1 read count in at least 20% of tumour samples in a given cancer type were considered to be expressed and included in this analysis. We first calculated the mean log2FC value of genes with Early_N_-to-Late_T_ timing in each cancer type. Next, the same number of genes that were early replicated in the tissue-of-origin were randomly sampled from expressed genes to calculate their mean log2FC. This step was repeated 100,000 times. The mean and 95% confidence interval of the mean log2FC values was constructed and compared to the observed mean log2FC. The empirical *p*-value was calculated by counting how many bootstrapped mean log2FC values of Late_N+T_ genes were greater than the observed mean values of Late_N_-to-Early_T_ genes divided by the total number of iterations, or equivalently, how many bootstrapped mean log2FC values of Early_N+T_ genes were lower than the observed mean values of Early_N_-to-Late_T_ genes divided by the total number of iterations.

To validate that differences in the expression were not driven by somatic copy number alterations (SCNAs), a similar bootstrapping method was applied to test for significant differences in copy number of genes with ART. Instead of using the log2FC per gene, the mean copy number relative to ploidy was considered. The mean copy number value per gene was calculated by overlapping the copy number segments of TCGA tumours with a certain gene and averaging the copy number relative to the overlapping size of the segments. For this, the total raw copy number values were used. Next, the difference between the mean copy number of a gene and the ploidy of the tumour was calculated and divided by the ploidy estimate to normalise the value between 0 and 1. This normalised difference per gene was provided to the bootstrapping method described above. The empirical *p*-value was calculated by counting how many bootstrapped mean values of Late_N+T_ genes were greater than the observed mean values of Late_N_-to-Early_T_ genes divided by the total number of iterations, or equivalently, how many bootstrapped mean values of Early_N+T_ genes were lower than the observed mean values of Early_N_-to-Late_T_ genes divided by the total number of iterations.

### Statistical analyses

All statistical tests were performed in the R statistical environment version ≥3.5.1 unless further stated. No statistical methods were used to predetermine the sample size. In general, comparisons between two groups were made using an unpaired two-sided Wilcoxon test. In instances where values of different RT groups within the same tumour were compared to each other, a paired Wilcoxon test was used. To examine the significance of the association between a certain feature and the RT classifications, a contingency table was created, and a two-sided Fisher’s exact test was applied. For all statistical analyses, the included data points were either plotted or annotated in the corresponding figure. In general, *p*-values were indicated as, ns: *p* ≥ 0.05; *p < 0.05; **p < 0.01; ***p < 0.001; ****p < 0.0001. In cases where the data is displayed as a box plot, the centre line represents the median value, the limits represent the 25th and 75th percentiles, and the whiskers extend from the box to the largest and lowest value no further than 1.5 * IQR away from the box, where IQR is the interquartile range.

### Reporting summary

Further information on research design is available in the Nature Portfolio Reporting Summary linked to this article.

### Supplementary information

Supplementary Information

Peer Review File

Reporting Summary

## Data Availability

Processed data to reproduce the analyses of this study including the replication timing signal data in 50 kb bins for the 31 cell lines analysed in this study can be accessed via Zenodo^82^. This repository does not include data from the Genomics England lung cohort due to restricted access. The Genomics England lung cohort is part of the 100,000 Genomes Project whose data are held in a secure research environment and are only available to registered users. For further information on how to obtain access visit https://www.genomicsengland.co.uk/research/academic. Somatic variants for the 560 WGS breast cancer dataset are available on the International Cancer Genome Consortium Data Portal (https://dcc.icgc.org/) and were retrieved via ftp://ftp.sanger.ac.uk/pub/cancer/Nik-ZainalEtAl-560BreastGenomes/. Supplementary files from Nik-Zainal et al.^2^ were downloaded for additional information, including clinical data. The TCGA data were retrieved through the database of Genotypes and Phenotypes (dbGaP) authorisation (accession no. phs000854.v3.p8). Information about TCGA and the investigators and institutions who constitute the TCGA research network can be found at https://cancergenome.nih.gov/. The accession numbers for the raw Repli-seq data of the 16 cell lines downloaded from ENCODE are listed in Supplementary Table 2. The accession numbers for the Hi-C data downloaded from ENCODE are provided in Supplementary Table 3. The raw data of the 13 in-house repli-sequenced cell lines has been made publicly available on SRA under the BioProject accession number PRJNA1096133. The raw data of the TRACERx PDCs (from the TRACERx study) used during this study has been deposited at the European Genome–phenome Archive (EGA), which is hosted by The European Bioinformatics Institute (EBI) and the Centre for Genomic Regulation (CRG) under the accession code EGAS00001007773 and is under controlled access due to its nature and commercial licences. Specifically, data is available through the Cancer Research UK & University College London Cancer Trials Centre (ctc.tracerx@ucl.ac.uk) for academic non-commercial research purposes only and is subject to review of a project proposal by the TRACERx data access committee, entering into an appropriate data access agreement and subject to any applicable ethical approvals. A response to the request for access is typically provided within 10 working days after the committee has received the relevant project proposal and all other required information. The access to the data will expire on the third anniversary of the effective date of the agreement.

## Code availability

The majority of statistical analyses were performed in the R statistical environment version ≥3.5.1. The code for the data analysis can be accessed via Zenodo^82^. The Repli-seq pipeline developed for this study is available for download from https://github.com/McGranahanLab/RepliSeqPipeline.
