# Supplementary material for: Replication timing alterations are associated with mutation acquisition during breast and lung cancer evolution
Source: Nat Commun. 2024 Jul 18;15:6039. doi: 10.1038/s41467-024-50107-4 (PMC11255325; doi:10.1038/s41467-024-50107-4)
Supplement: Supplementary file 1 — Supplementary Information [file 41467_2024_50107_MOESM1_ESM.pdf]

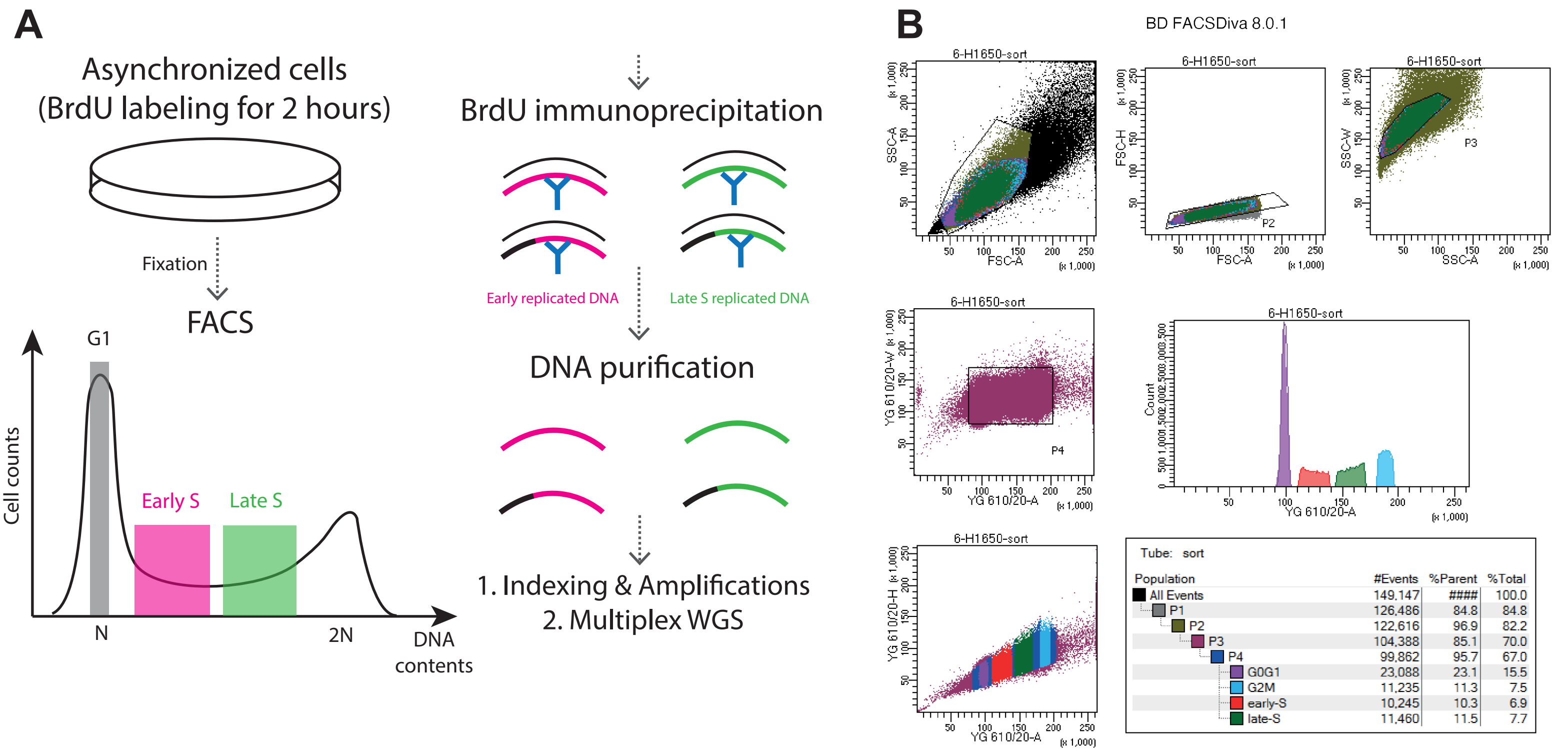

Supplementary Figure 1. The workflow of replication timing sequencing (Repli-seq) protocol. A Asynchronized cells in cell culturing were labeled with bromodeoxyuridine 5-bromo-2'-deoxyuridine (BrdU) for 2 hours to label nascently replicated DNA fragments. After fixation, BrdU labeled cells were sorted into early or late replicated samples based on the amount of propidium iodide (PI) stained DNA contents using fluorescence-activated cell sorting (FACS). BrdU immunoprecipitation was then performed to precipitate early or late replicated DNA regions. After DNA purification, multiplex whole genome sequencing (WGS) was conducted to generate fastq files of early or late replicated sequences for further analyses. **B** The gating strategy during FACS sorting for the Repli-seq protocol applied in this study. The sorting of H1650, a lung adenocarcinoma (LUAD) cell line, is shown as an example. In brief, cells were initially gated using forward scatter (FSC) versus side scatter (SSC) to exclude debris and aggregates. Subsequently, sequential gating strategies were employed to identify single cells, including gating based on FSC-A versus FSC-H, SSC-A versus SSC-W, and propidium iodide (PI)-A versus PI-W to further exclude apoptotic cells and doublets. The G0/G1 and G2/M phases were distinguished by the peak of PI staining intensity, incorporating DNA content with cell size (FSC-A) as a reference. To ensure accuracy, early-S and late-S phases were equally gated between the G0/G1 and G2/M phases, while the edges of each population were avoided to minimize potential artifacts. More details can be found in the Methods section.

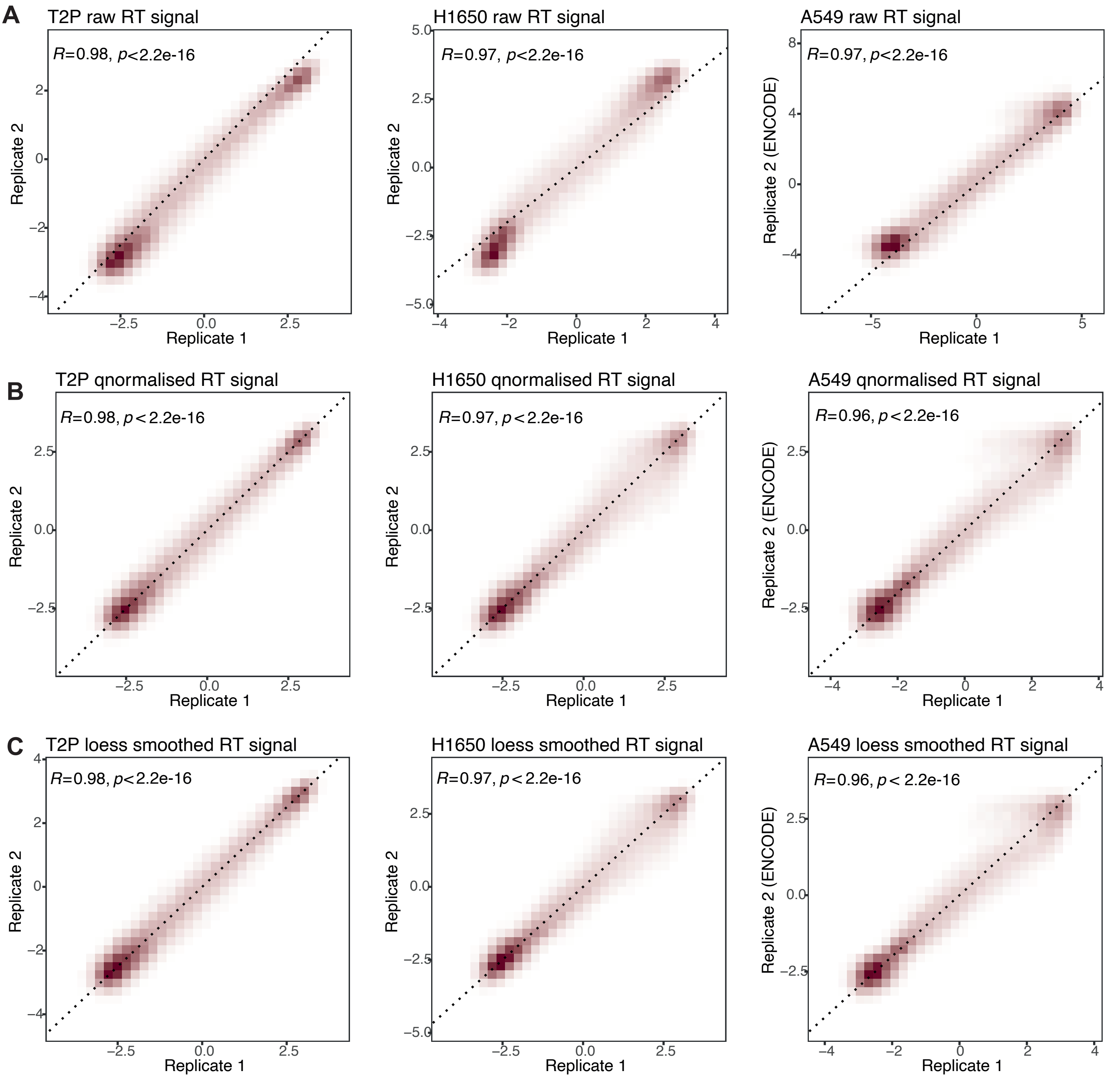

Supplementary Figure 2. Validation of replication timing signals in 50 kb windows using biological replicates and its association with copy number alterations in cancer. The replicates of T2P and H1650 were obtained by applying our IN-STUDY Repli-seq protocol to both replicates. One replicate of A549 was produced by applying our IN-STUDY Repli-seq protocol whereas the other one was downloaded from ENCODE. 2D density plots of raw replication timing (RT) values (**A**), quantile normalised RT values (**B**) and loess smoothed RT values (**C**) between two replicates of these three different cell lines are shown, presenting their Pearson correlation test values (R and p values). The loess smoothed RT values were used as RT signals in our further analyses. **D** The association between copy number alterations and RT signals within each of BRCA and LUAD cell lines. Median RT signals and standard deviation of bootstrapped bins per copy number status were adjusted for differences in the size of genomic regions with gained, lost or no (neutral) copy number alterations.

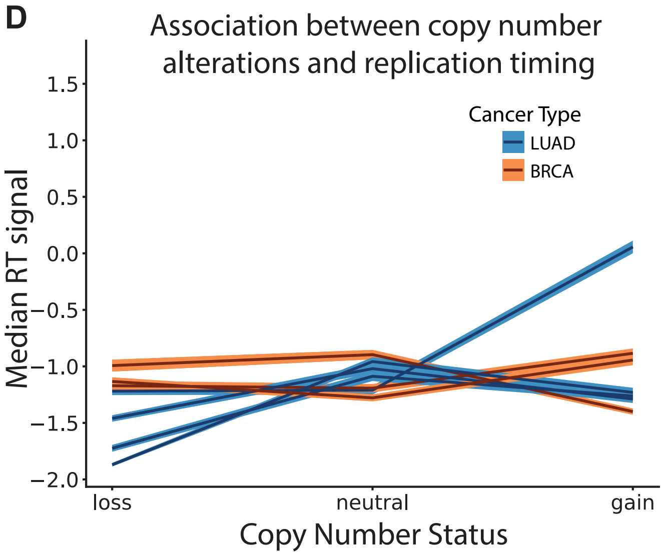

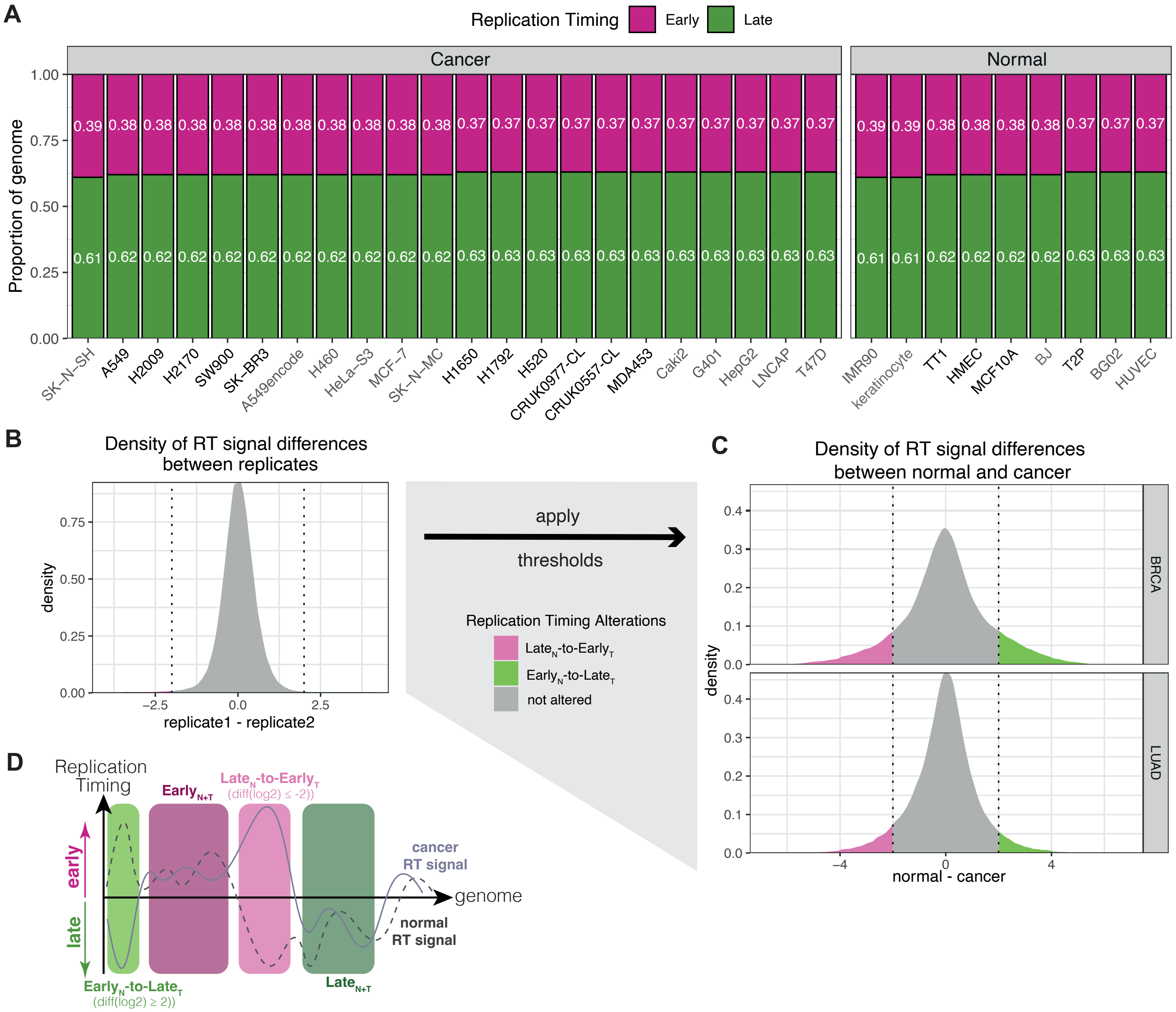

Supplementary Figure 3. Methods to define replication timing and altered replication timing across the genome. **A** Proportions of early and late replicated regions across the genome for all 31 cell lines. Cell lines highlighted in grey were downloaded from ENCODE (N=16) and cell line names coloured in black are part of our IN-STUDY dataset (N=15). **B** Density of differences in the replication timing (RT) signal between replicates of the T2P, H1650 and A549 cell line. The dotted black line shows the threshold of |2| to identify altered replication timing (ART) which represents the rounded values of the 99% confidence interval of the displayed distribution. **C** Density of differences between cancer cell lines and their matched tissue-of-origin with the application of thresholds to identify ART regions per cancer type. **D** Cartoon to illustrate the different definitions of ART and unaltered RT classified and investigated in this study.

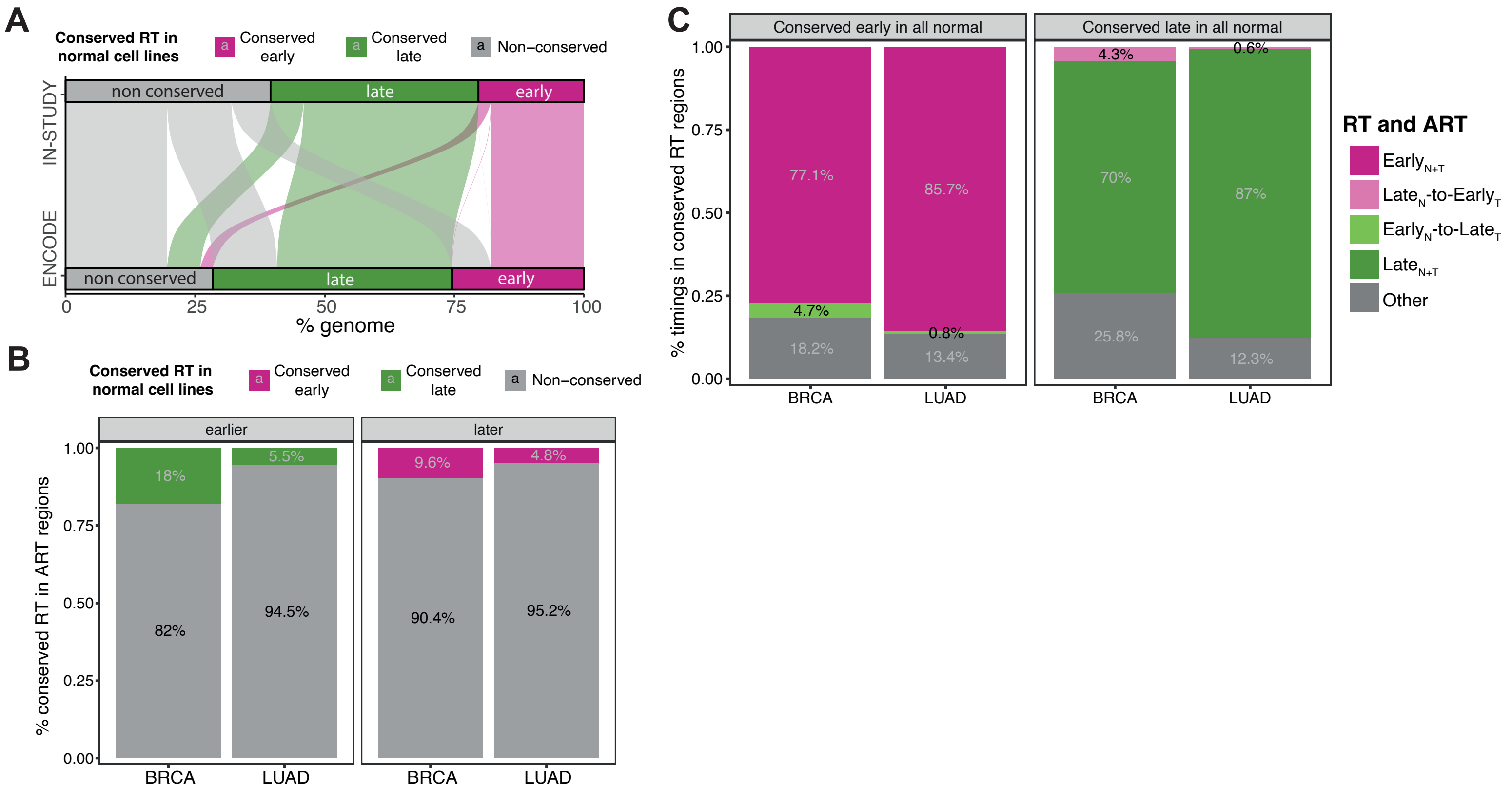

Supplementary Figure 4. Comparing conserved RT regions in non-malignant cell lines with ART regions identified in cancer. A Comparing the conserved early and late replication timing (RT) regions identified in 4 IN-STUDY versus 5 ENCODE non-malignant ("normal") cell lines. **B** Proportions of conserved early or late replicated regions among altered replication timing (ART) regions within each cancer type. **C** Proportions of unaltered RT and ART regions in cancer among conserved early or late RT regions identified in all 9 normal cell lines.

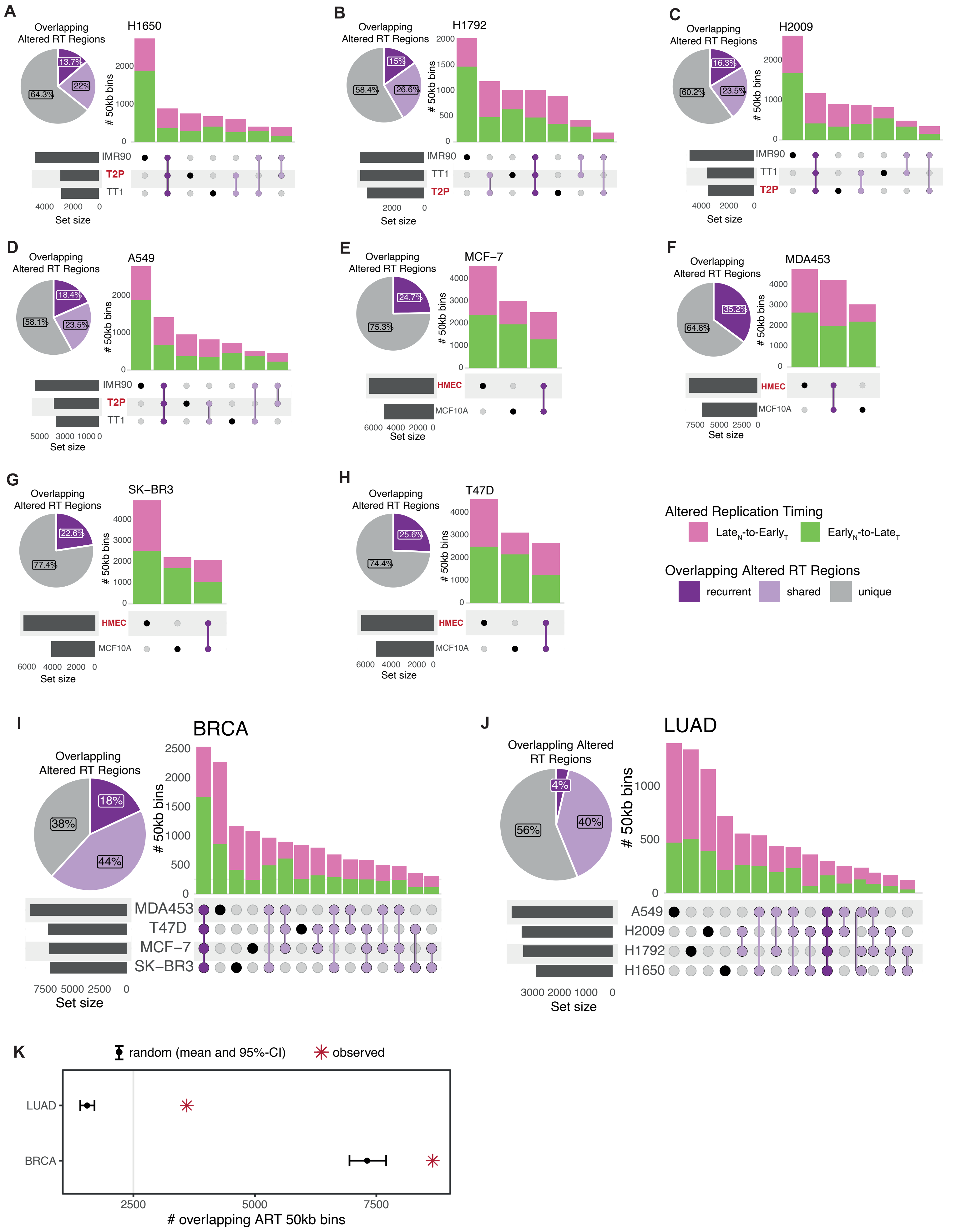
A
Overlapping Altered RT Regions
H1650
B
H1792
C
H2009
D
A549
E
MCF−7
F
MDA453
G
SK−BR3
H
T47D
IMR90
T2P
TT1
HMEC
MCF10A
Set size
50kb bins
Altered Replication Timing
Late_N-to-Early_T
Early_N-to-Late_T
Overlapping Altered RT Regions
recurrent
shared
unique
I
BRCA
J
LUAD
Overlapping Altered RT Regions
MDA453
T47D
MCF−7
SK−BR3
A549
H2009
H1792
H1650
K
random (mean and 95%-CI)
observed
LUAD
BRCA
overlapping ART 50kb bins

Supplementary Figure 5. Comparisons of ART regions in cancer cell lines of the same cancer type and when using the wrong normal as reference. A-H Each panel includes an upset plot presenting the number of overlapping 50 kb bins with altered replication timing (ART) when using three different normal lung cell lines (**A-D**) or using two different normal breast cell lines (**E-H**) as reference. The reported tissue-of-origins (T2P for lung adenocarcinomas (LUAD), and HMEC for breast carcinomas (BRCA)) are highlighted in red. A pie chart is added to each panel to display the fraction of bins that have been identified as ART with all three normal cell lines (recurrent), with two normal cell lines (shared) and only with one normal cell line (unique). **I-J** Upset plots showing the detailed breakdown of overlapping ART regions among four BRCA cell lines compared to HMEC as a normal reference (**I**) or among four LUAD cell lines compared to T2P (**J**). **K** Results of the bootstrapping method to test whether the number of overlapping (shared + recurrent) ART bins is significantly higher than random. For each cancer type, ART domains within each cell line were randomly distributed and overlaps were calculated 1000 times. The mean number of overlapping 50 kb bins with ART within the randomly created dataset are displayed in black whereas the observed number is highlighted as red stars.

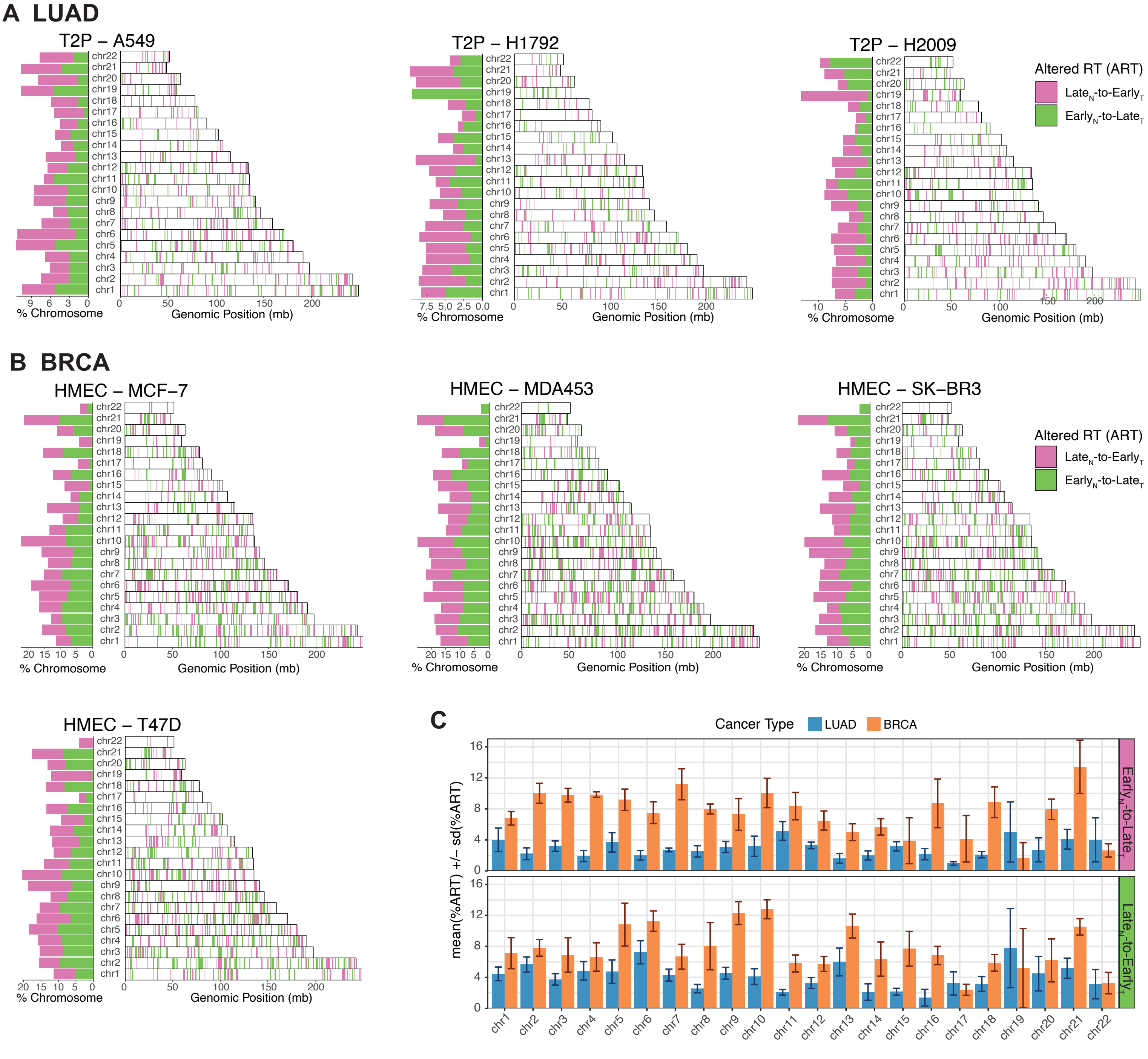

Supplementary Figure 6. Distribution of ART regions across the genome in cancer cell lines. A Heatmaps of the genomic locations of altered replication timing (ART) regions in three different lung adenocarcinoma (LUAD) cell lines (H1792, H2009 and A549) relative to its matching tissue-of-origin T2P. **B** Heatmaps of the genomic locations of ART regions in four different breast carcinoma (BRCA) cell lines (MCF-7, MDA453, SK-BR3 and T47D) relative to its matching tissue-of-origin HMEC. **C** Fractions of genomic regions with ART per chromosome and per cancer type (y-axis represents the mean fraction across cell lines within the same cancer type and the length of error bars shows two times the standard deviation).

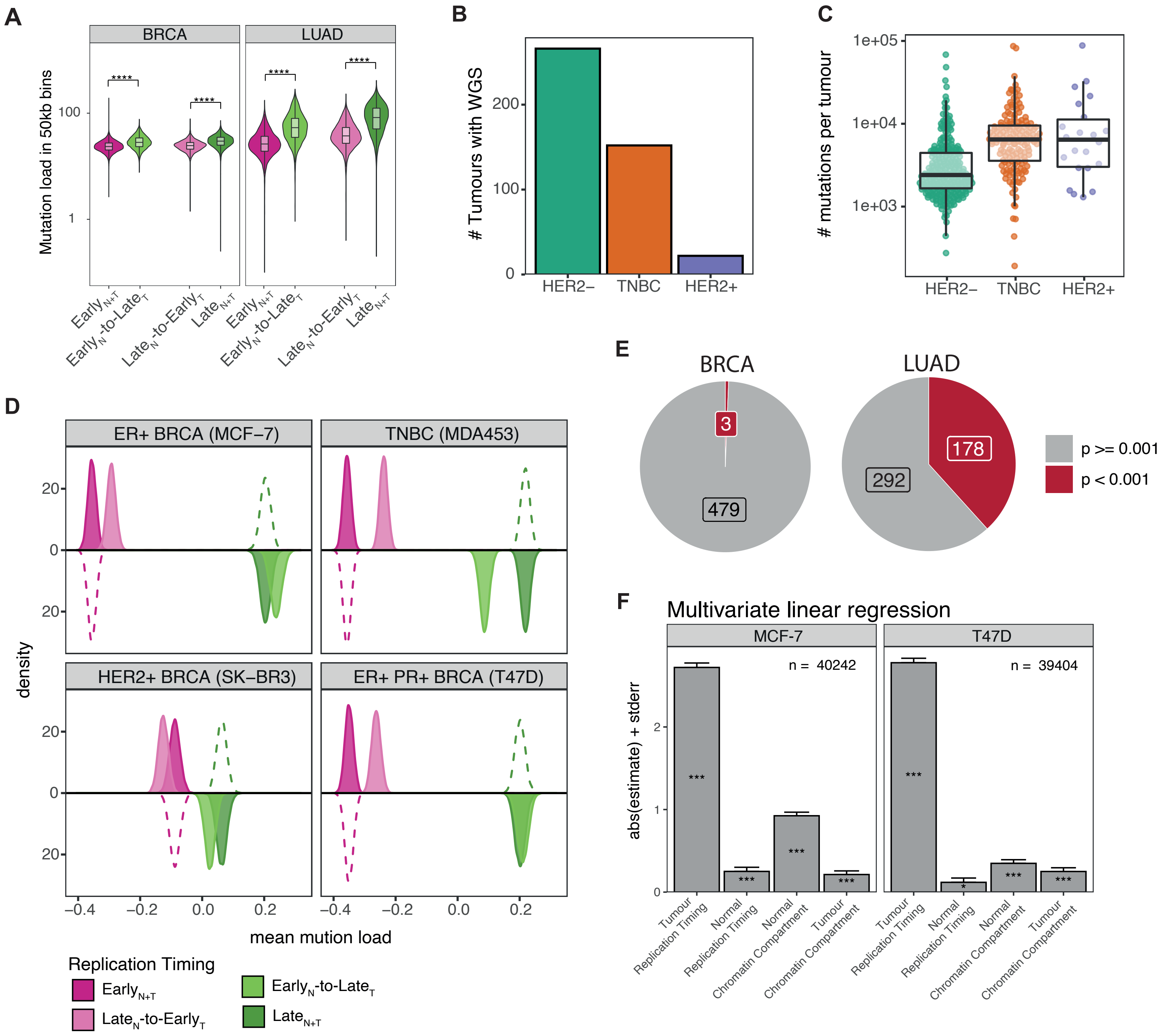

Supplementary Figure 7. The mutation load and mutation distribution in unaltered RT and ART regions in cancer cell lines. A Comparing the mutation load in unaltered replication timing (RT) and altered replication timing (ART) regions in breast carcinoma (BRCA) and lung adenocarcinoma (LUAD) tumours (Wilcoxon test, ns: p-value ≥ 0.05, *: p-value < 0.05, **: p-value < 0.01, ***: p-value < 0.001, ****: p-value < 0.0001). The centre line of the box plot represents the median value, the limits represent the 25th and 75th percentile, and the whiskers extend from the box to the largest and lowest value no further than 1.5 * IQR (interquartile range) away from the box. **B** Bar plot showing the number of tumours included in the BRCA whole genome sequencing (WGS) dataset classified as the different breast cancer subtypes. **C** Boxplots showing the number of mutations per tumour for the different BRCA subtypes. The centre line of the box plot represents the median value, the limits represent the 25th and 75th percentile, and the whiskers extend from the box to the largest and lowest value no further than 1.5 * IQR away from the box. **D** Distribution of the bootstrapped mean mutation load values in unaltered RT and ART regions in each BRCA subtype. The analysis of unaltered RT and ART regions in each BRCA cell line and the matched WGS data from tumours with the same BRCA subtype were used to estimate the mean mutation load distributions for the different breast cancer subtypes. **E** Pie charts representing the proportions of BRCA and LUAD tumours with a bootstrapping p value < 0.001 and therefore a significant difference in mean mutation load between unaltered $Early_{N+T}$ and $Late_{N+T}$ replicated regions. The annotated numbers show the total number of tumours with a bootstrapping p-value < 0.001 or >= 0.001. **F** Results of a multivariate regression model with BRCA mutation load as independent variable and z-transformed chromatin and replication timing signals in normal (HMEC) and tumour (MCF-7 or T47D) as dependent variables. Only genomic bins for which chromatin and replication timing signals were available in normal and tumour were considered (MCF-7: n = 40242; T47F: n = 39404). The bars represent the absolute values of the estimates representing the effect size of the different variables with the standard error added as error bar on top. (Coefficient Wald test, ns: p-value ≥ 0.05, *: p-value < 0.05, **: p-value < 0.01, ***: p-value < 0.001, ****: p-value < 0.0001).

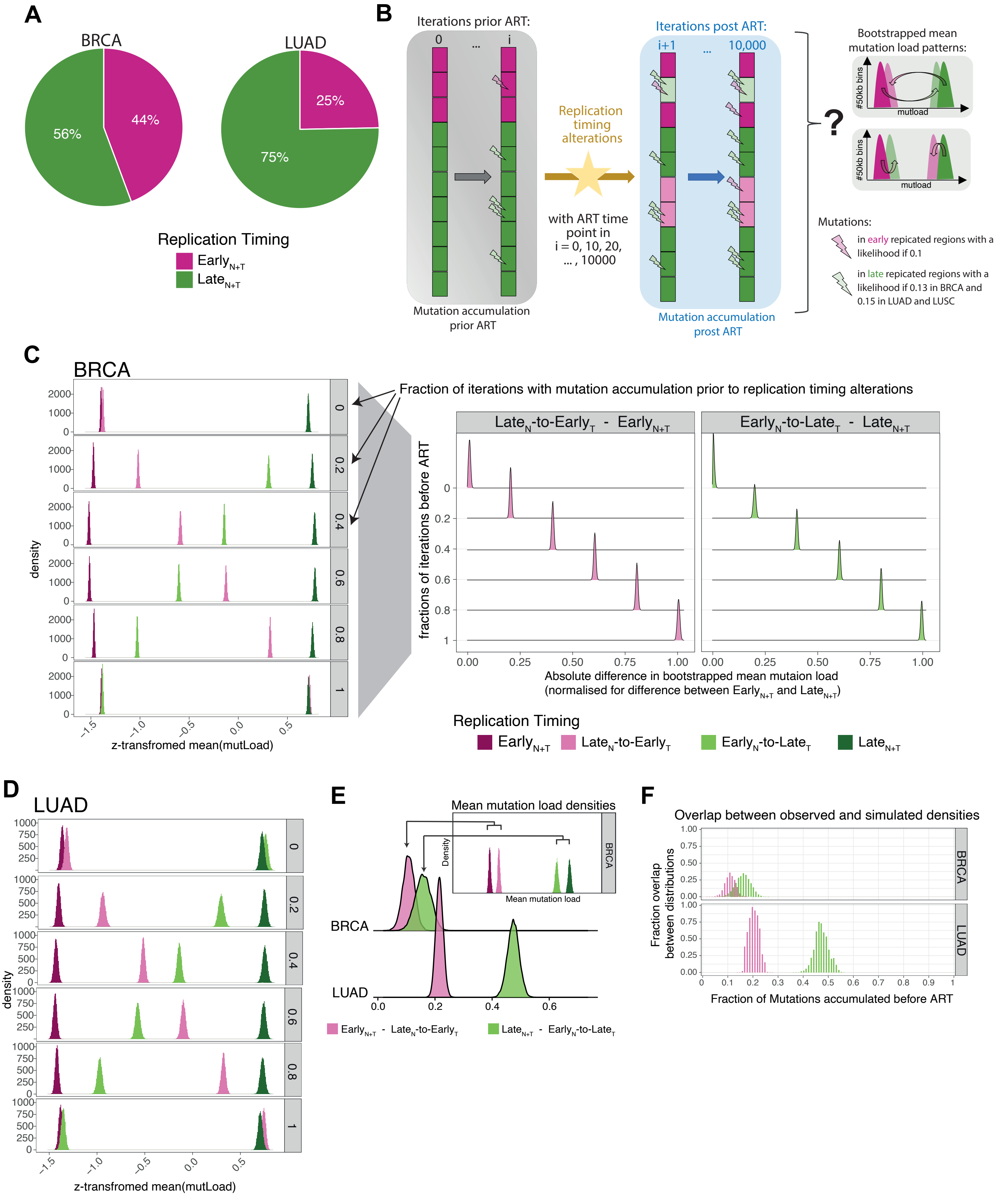
A
BRCA
LUAD
56%
44%
25%
75%
Replication Timing
Early N+T
Late N+T
B
Iterations prior ART:
Mutation accumulation prior ART
Replication timing alterations
with ART time point in i = 0, 10, 20, ... , 10000
Iterations post ART:
i+1 ... 10,000
Mutation accumulation post ART
Bootstrapped mean mutation load patterns:
#50kb bins
mutload
Mutations:
in early repicated regions with a likelihood if 0.1
in late repicated regions with a likelihood if 0.13 in BRCA and 0.15 in LUAD and LUSC
C
BRCA
density
z-transfromed mean(mutLoad)
Fraction of iterations with mutation accumulation prior to replication timing alterations
Late N-to-Early T - Early N+T
Early N-to-Late T - Late N+T
fractions of iterations before ART
Absolute difference in bootstrapped mean mutaion load
(normalised for difference between Early N+T and Late N+T)
Replication Timing
Early N+T
Late N-to-Early T
Early N-to-Late T
Late N+T
D
LUAD
density
z-transfromed mean(mutLoad)
E
Mean mutation load densities
Density
Mean mutation load
BRCA
LUAD
Early N+T - Late N-to-Early T
Late N+T - Early N-to-Late T
F
Overlap between observed and simulated densities
Fraction overlap between distributions
Fraction of Mutations accumulated before ART
BRCA
LUAD

Supplementary Figure 8. Simulations of different time points of ART relative to the mutation accumulation in the MRCA. **A** Fraction of mutations per Mb in $Early_{N+T}$ and $Late_{N+T}$ replicated regions when only considering mutations in unaltered replication timing (RT) regions. **B** Workflow of simulations to investigate the timing of altered replication timing (ART) occurrence. **C-D** Mean mutation load distributions as result from a subset of simulations for breast carcinomas (BRCA) (**C**) and lung adenocarcinomas (LUAD) (**D**). The relative differences between mean mutations load values in unaltered RT and ART regions were calculated and used to compare the expected and observed mutation distribution patterns. **E** Observed distributions of the relative difference between the mean mutation load densities between unaltered $Early_{N+T}$ and $Late_N$-to-$Early_T$ replicated regions (light pink) and between unaltered $Late_{N+T}$ and $Early_N$-to-$Late_T$ replicated regions (light green). **F** Proportion of overlapping values between the simulated and the observed distributions of absolute differences in bootstrapped mean mutation load values between unaltered RT and ART regions to estimate the evolutionary timing of replication timing alterations relative to mutation accumulation in BRCA and LUAD.

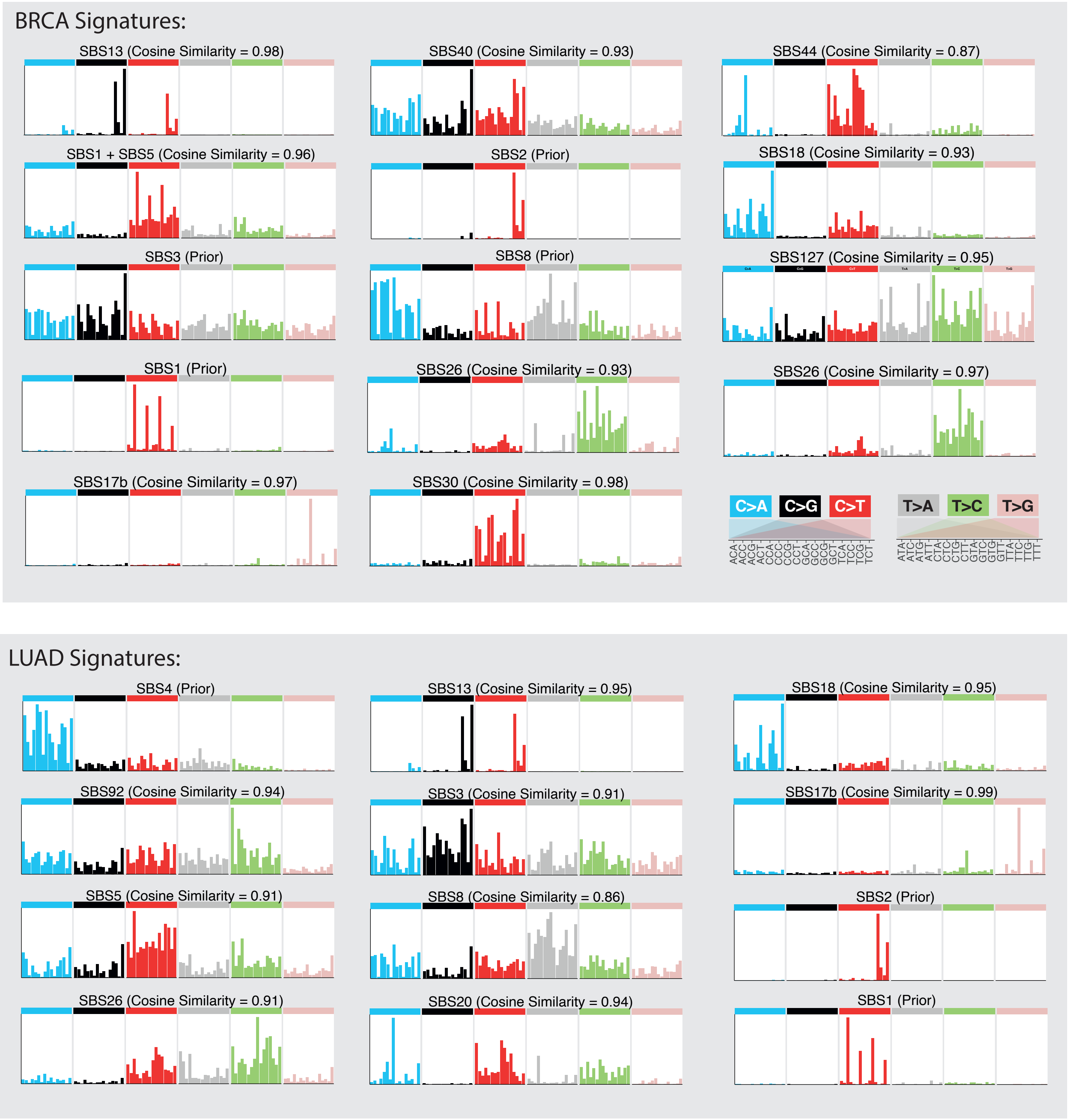

Supplementary Figure 9. Profiles of mutational signatures identified in unaltered replication timing and altered replication timing regions in breast carcinoma (BRCA) and lung adenocarcinoma (LUAD) tumours. The x-axis represents the 96-trinucleotide context of the mutations ordered as displayed in the key. The y-axis represents the fraction of single base subsitutions (SBSs) per trinucleotide context. The axis labels were excluded per plot due to space restrictions. Signatures that were identified to be commonly active in lung and breast cancer were used as priors in the de novo signature extraction algorithm and are labelled in this Figure accordingly. For further details on the algorithm and the selection of priors see the Methods section.

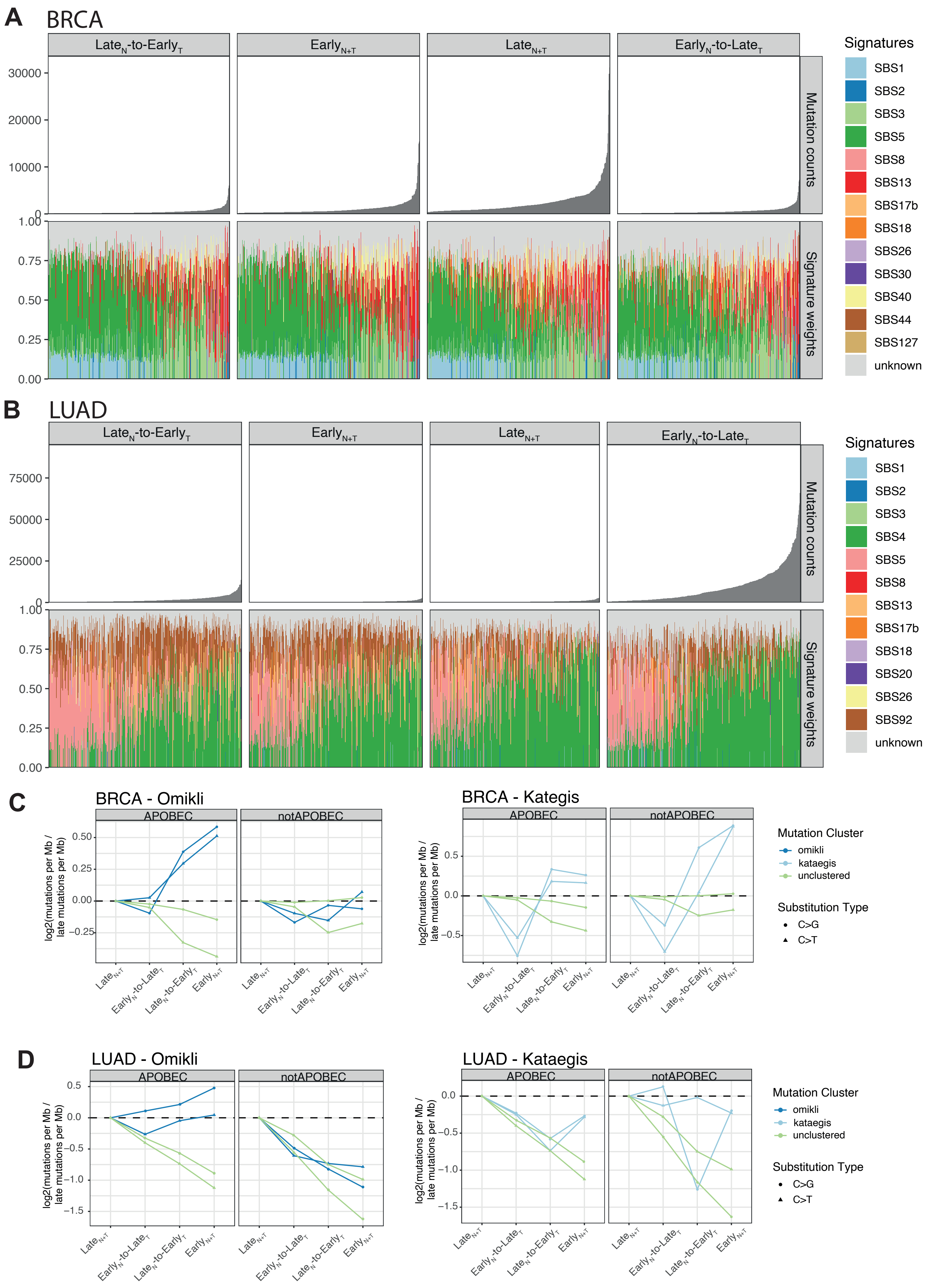
A
BRCA
Late$_N$-to-Early$_T$
Early$_{N+T}$
Late$_{N+T}$
Early$_N$-to-Late$_T$
Mutation counts
Signature weights
30000
20000
10000
0
1.00
0.75
0.50
0.25
0.00
Signatures
SBS1
SBS2
SBS3
SBS5
SBS8
SBS13
SBS17b
SBS18
SBS26
SBS30
SBS40
SBS44
SBS127
unknown
B
LUAD
Late$_N$-to-Early$_T$
Early$_{N+T}$
Late$_{N+T}$
Early$_N$-to-Late$_T$
Mutation counts
Signature weights
75000
50000
25000
0
1.00
0.75
0.50
0.25
0.00
Signatures
SBS1
SBS2
SBS3
SBS4
SBS5
SBS8
SBS13
SBS17b
SBS18
SBS20
SBS26
SBS92
unknown
C
BRCA - Omikli
APOBEC
notAPOBEC
log2(mutations per Mb / late mutations per Mb)
0.50
0.25
0.00
−0.25
Late$_{N+T}$
Early$_N$-to-Late$_T$
Late$_N$-to-Early$_T$
Early$_{N+T}$
BRCA - Kategis
APOBEC
notAPOBEC
0.5
0.0
−0.5
Mutation Cluster
omikli
kataegis
unclustered
Substitution Type
C>G
C>T
D
LUAD - Omikli
APOBEC
notAPOBEC
log2(mutations per Mb / late mutations per Mb)
0.5
0.0
−0.5
−1.0
−1.5
Late$_{N+T}$
Early$_N$-to-Late$_T$
Late$_N$-to-Early$_T$
Early$_{N+T}$
LUAD - Kataegis
APOBEC
notAPOBEC
0.0
−0.5
−1.0
−1.5
Mutation Cluster
omikli
kataegis
unclustered
Substitution Type
C>G
C>T

Supplementary Figure 10. Exposures of mutational signatures and clustered APOBEC3 mutations identified in unaltered RT and ART regions in BRCA and LUAD. A-B Exposures of signatures inferred by the hierarchical Dirichlet process (HDP) approach in different unaltered replication timing (RT) and altered replication timing (ART) regions which were assigned to known signatures in breast carcinoma (BRCA) (A) and lung adenocarcinoma (LUAD) (B) tumours. **C-D** Mutation rates per Mb in different unaltered RT and ART regions relative to unaltered $Late_{N+T}$ replicated regions for omikli (small clustered APOBEC3 mutations), kataegis (clustered APOBEC3 mutations) and unclustered mutations in BRCA (**C**) and LUAD (**D**) tumours. The left panel in each plot displays the results of APOBEC induced mutations, and the right shows the results of non-APOBEC related mutations as a control.

Supplementary Table 1. Cancer cell lines and corresponding non-malignant cell lines derived from the matched tissue-of-origin for which the Repli-seq protocol was performed in this study.

Cell line	Replicates	Repli-WGS coverage	Data source	Disease type	Description
HMEC	x 1	12 samples per lane	IN-STUDY	Normal (derived from tissue-of-origin for BRCA)	Human mammary epithelial cells
MCF10A	x 1	12 samples per lane	IN-STUDY	Fibrocystic disease	
SK-BR3	x 1	12 samples per lane	IN-STUDY	BRCA	HER2 positive breast cancer cells
MDA453	x 1	12 samples per lane	IN-STUDY	BRCA	Triple negative breast cancer cells
MCF-7	ENCODE	/	ENCODE	BRCA	ER positive breast cancer cells
T47D	ENCODE	/	ENCODE	BRCA	ER and PR positive breast cancer cells
T2P	x 2	12 or 6 samples per lane	IN-STUDY	Normal (derived from tissue-of-origin for LUAD)	Type 2 pneumocytes
TT1	x 1	12 samples per lane	IN-STUDY	Normal	Type 1 like pneumocytes
A549	x 1 (+ ENCODE)	12 samples per lane	IN-STUDY	LUAD	
H1650	x 2	12 or 6 samples per lane	IN-STUDY	LUAD	
H1792	x 1	12 samples per lane	IN-STUDY	LUAD	
H2009	x 1	12 samples per lane	IN-STUDY	LUAD	
CRUK0557-CL	x 1	6 samples per lane	IN-STUDY	PDCs derived from TRACERx LUAD tumour	
CRUK0977-CL	x 1	6 samples per lane	IN-STUDY	PDCs derived from TRACERx LUAD tumour	
H2170	x 1	12 samples per lane	IN-STUDY	LUSC	

H520	x 1	12 samples per lane	IN-STUDY	LUSC	
SW900	x 1	12 samples per lane	IN-STUDY	LUSC	

Supplementary Table 2. Accession numbers of the Repli-Seq data downloaded from ENCODE.

File accession	Experiment accession	Biosample term id	Biosample term name	Biosample phase
ENCFF692XJH	ENCSR869SPM	EFO:0002150	Caki2	late S phase
ENCFF884BCM	ENCSR169UZM	EFO:0003044	NCI-H460	late S phase
ENCFF029RFD	ENCSR594FTB	EFO:0001086	A549	late S phase
ENCFF609OGL	ENCSR322LSA	EFO:0001247	T47D	late S phase
ENCFF201QCJ	ENCSR291SBE	EFO:0002860	SK-N-MC	late S phase
ENCFF847QAU	ENCSR801ISP	EFO:0002860	SK-N-MC	early S phase
ENCFF001GIF	ENCSR305ALH	EFO:0002778	BG02	S3 phase
ENCFF001GPS	ENCSR441OPI	EFO:0002791	HeLa-S3	S4 phase
ENCFF001GRC	ENCSR738PPH	CL:0002618	HUVEC	S3 phase
ENCFF001GSY	ENCSR338BMC	EFO:0002067	K562	S2 phase
ENCFF001GSC	ENCSR810JIM	EFO:0002067	K562	G2 phase
ENCFF001GSD	ENCSR810JIM	EFO:0002067	K562	G2 phase
ENCFF001GVB	ENCSR016CNN	EFO:0003072	SK-N-SH	G2 phase
ENCFF001GRB	ENCSR420LJO	CL:0002618	HUVEC	S1 phase
ENCFF001GOT	ENCSR098AZD	EFO:0002791	HeLa-S3	G2 phase
ENCFF001GPL	ENCSR911SCA	EFO:0001187	HepG2	S1 phase
ENCFF001GPM	ENCSR911SCA	EFO:0001187	HepG2	S1 phase
ENCFF001GRP	ENCSR554HRW	EFO:0001196	IMR-90	S1 phase
ENCFF001GRT	ENCSR903LKJ	EFO:0001196	IMR-90	S2 phase
ENCFF001GVC	ENCSR534FPJ	EFO:0003072	SK-N-SH	S1 phase
ENCFF001GVA	ENCSR000CXA	EFO:0003072	SK-N-SH	G1b phase
ENCFF001GIR	ENCSR000CXO	EFO:0002779	BJ	G1b phase
ENCFF001GIS	ENCSR000CXO	EFO:0002779	BJ	G1b phase
ENCFF748HXM	ENCSR787TGS	EFO:0002179	G401	late S phase
ENCFF897XSM	ENCSR385QAX	EFO:0002071	LNCAP	early S phase
ENCFF111ERU	ENCSR277AIN	EFO:0002179	G401	early S phase
ENCFF435OVV	ENCSR660KEM	EFO:0001086	A549	early S phase
ENCFF326XAE	ENCSR129TEN	EFO:0003044	NCI-H460	early S phase
ENCFF377MRQ	ENCSR868TAH	EFO:0002150	Caki2	early S phase
ENCFF174OOB	ENCSR331WAS	EFO:0001247	T47D	early S phase
ENCFF583YFW	ENCSR089VDE	EFO:0002071	LNCAP	late S phase
ENCFF001GRK	ENCSR193NLV	CL:0002618	HUVEC	S4 phase
ENCFF001GJY	ENCSR852QFP	EFO:0002779	BJ	S4 phase

ENCFF001GLD	ENCSR852QFP	EFO:0002779	BJ	S4 phase
ENCFF001GPP	ENCSR835JVR	EFO:0001187	HepG2	S2 phase
ENCFF001GPQ	ENCSR835JVR	EFO:0001187	HepG2	S2 phase
ENCFF001GPV	ENCSR345QON	EFO:0001187	HepG2	S3 phase
ENCFF001GPW	ENCSR345QON	EFO:0001187	HepG2	S3 phase
ENCFF001GPT	ENCSR628LNL	EFO:0002791	HeLa-S3	S3 phase
ENCFF001GUH	ENCSR000CXC	CL:0000312	keratinocyte	G1b phase
ENCFF001GUE	ENCSR407DUT	CL:0000312	keratinocyte	G2 phase
ENCFF001GVO	ENCSR512PWK	EFO:0003072	SK-N-SH	S2 phase
ENCFF001GTA	ENCSR589KVH	EFO:0002067	K562	S3 phase
ENCFF001GTB	ENCSR802MDS	EFO:0002067	K562	S4 phase
ENCFF001GQI	ENCSR331IKA	CL:0002618	HUVEC	G2 phase
ENCFF001GQJ	ENCSR331IKA	CL:0002618	HUVEC	G2 phase
ENCFF001GQG	ENCSR000CXG	EFO:0001187	HepG2	G1b phase
ENCFF001GIG	ENCSR999NSK	EFO:0002778	BG02	S4 phase
ENCFF001GJM	ENCSR075IID	EFO:0002779	BJ	S3 phase
ENCFF001GJS	ENCSR075IID	EFO:0002779	BJ	S3 phase
ENCFF001GIY	ENCSR935ULX	EFO:0002779	BJ	S1 phase
ENCFF001GJE	ENCSR935ULX	EFO:0002779	BJ	S1 phase
ENCFF001GQY	ENCSR000CXI	CL:0002618	HUVEC	G1b phase
ENCFF001GRY	ENCSR000CXE	EFO:0002067	K562	G1b phase
ENCFF001GRZ	ENCSR000CXE	EFO:0002067	K562	G1b phase
ENCFF001GOU	ENCSR000CXH	EFO:0002791	HeLa-S3	G1b phase
ENCFF001GHI	ENCSR347TZI	EFO:0002778	BG02	G2 phase
ENCFF001GHJ	ENCSR347TZI	EFO:0002778	BG02	G2 phase
ENCFF001GIE	ENCSR226MSR	EFO:0002778	BG02	S2 phase
ENCFF001GTM	ENCSR170QBY	EFO:0001203	MCF-7	S2 phase
ENCFF001GTX	ENCSR831UBH	EFO:0001203	MCF-7	S4 phase
ENCFF001GUJ	ENCSR886ISU	CL:0000312	keratinocyte	S1 phase
ENCFF001GUK	ENCSR406MAP	CL:0000312	keratinocyte	S3 phase
ENCFF001GTW	ENCSR404GFT	EFO:0001203	MCF-7	S3 phase
ENCFF001GUX	ENCSR340MHM	CL:0000312	keratinocyte	S4 phase
ENCFF001GVT	ENCSR552ZRB	EFO:0003072	SK-N-SH	S4 phase
ENCFF001GOV	ENCSR789SBW	EFO:0002791	HeLa-S3	S2 phase
ENCFF001GQU	ENCSR772AGI	CL:0002618	HUVEC	S2 phase
ENCFF001GSM	ENCSR874RPM	EFO:0001196	IMR-90	S3 phase
ENCFF001GSG	ENCSR958IUL	EFO:0002067	K562	S1 phase
ENCFF001GSH	ENCSR958IUL	EFO:0002067	K562	S1 phase
ENCFF001GOX	ENCSR811MTR	EFO:0002791	HeLa-S3	S1 phase
ENCFF001GTN	ENCSR727ZRP	EFO:0001203	MCF-7	S1 phase
ENCFF001GRN	ENCSR000CXF	EFO:0001196	IMR-90	G1b phase
ENCFF001GHR	ENCSR000CXB	EFO:0002778	BG02	G1b phase
ENCFF001GHU	ENCSR537ICK	EFO:0002778	BG02	S1 phase
ENCFF001GUI	ENCSR769UFO	CL:0000312	keratinocyte	S2 phase

ENCFF001GTJ	ENCSR292KPN	EFO:0001203	MCF-7	G2 phase
ENCFF001GVP	ENCSR885JHI	EFO:0003072	SK-N-SH	S3 phase
ENCFF001GPY	ENCSR109NSK	EFO:0001187	HepG2	S4 phase
ENCFF001GPZ	ENCSR109NSK	EFO:0001187	HepG2	S4 phase
ENCFF001GRO	ENCSR333KJW	EFO:0001196	IMR-90	G2 phase
ENCFF001GIU	ENCSR895MIM	EFO:0002779	BJ	G2 phase
ENCFF001GIV	ENCSR895MIM	EFO:0002779	BJ	G2 phase
ENCFF001GJJ	ENCSR750HRE	EFO:0002779	BJ	S2 phase
ENCFF001GJK	ENCSR750HRE	EFO:0002779	BJ	S2 phase
ENCFF001GSL	ENCSR816UHB	EFO:0001196	IMR-90	S4 phase
ENCFF001GPG	ENCSR216KAB	EFO:0001187	HepG2	G2 phase
ENCFF001GPH	ENCSR216KAB	EFO:0001187	HepG2	G2 phase
ENCFF001GTI	ENCSR000CXD	EFO:0001203	MCF-7	G1b phase

Supplementary Table 3. Accession numbers of the Hi-C data downloaded from ENCODE including the ENCODE pipeline version that the data was processed with.

Cell type	Hi-C protocol	Experiment	Contact matrix	Pipeline
MCF-7	intact	ENCSR660LPJ	ENCFF420JTA	v1.10.0
HMEC	intact	ENCSR707XVJ	ENCFF512PQA	v1.11.2
T47D	in situ	ENCSR549MGQ	ENCFF832RTC	v1.10.0
HMEC	in situ	ENCSR711AVS	ENCFF943JRY	v1.15.1
A549	in situ	ENCSR444WCZ	ENCFF689CUX	v1.10.0
