# Supplementary material for: Replication timing alterations are associated with mutation acquisition during breast and lung cancer evolution
Source: Nat Commun. 2024 Jul 18;15:6039. doi: 10.1038/s41467-024-50107-4 (PMC11255325; doi:10.1038/s41467-024-50107-4)
Supplement: Supplementary file 3 — Reporting Summary [file 41467_2024_50107_MOESM3_ESM.pdf]

nature portfolio

Corresponding author(s): Nicholas McGranahan, Nnennaya Kanu

Last updated by author(s): Jun 25, 2024

Reporting Summary

Nature Portfolio wishes to improve the reproducibility of the work that we publish. This form provides structure for consistency and transparency in reporting. For further information on Nature Portfolio policies, see our Editorial Policies and the Editorial Policy Checklist.

Statistics

For all statistical analyses, confirm that the following items are present in the figure legend, table legend, main text, or Methods section.

n/a	Confirmed	
☐	☒	The exact sample size (*n*) for each experimental group/condition, given as a discrete number and unit of measurement
☐	☒	A statement on whether measurements were taken from distinct samples or whether the same sample was measured repeatedly
☐	☒	The statistical test(s) used AND whether they are one- or two-sided<br>*Only common tests should be described solely by name; describe more complex techniques in the Methods section.*
☐	☒	A description of all covariates tested
☐	☒	A description of any assumptions or corrections, such as tests of normality and adjustment for multiple comparisons
☐	☒	A full description of the statistical parameters including central tendency (e.g. means) or other basic estimates (e.g. regression coefficient) AND variation (e.g. standard deviation) or associated estimates of uncertainty (e.g. confidence intervals)
☐	☒	For null hypothesis testing, the test statistic (e.g. *F*, *t*, *r*) with confidence intervals, effect sizes, degrees of freedom and *P* value noted<br>*Give P values as exact values whenever suitable.*
☒	☐	For Bayesian analysis, information on the choice of priors and Markov chain Monte Carlo settings
☒	☐	For hierarchical and complex designs, identification of the appropriate level for tests and full reporting of outcomes
☐	☒	Estimates of effect sizes (e.g. Cohen's *d*, Pearson's *r*), indicating how they were calculated

Our web collection on statistics for biologists contains articles on many of the points above.

Software and code

Policy information about availability of computer code

Data collection	No software was used to collect data. The in-house Repli-Seq data was processed through our pipeline published on github https://github.com/McGranahanLab/RepliSeqPipeline. Other data was downloaded from public sources which are listed in our data availability statement.
Data analysis	R (version 3.5.1 and 4.0.2)<br>Python (version 3.9.5)<br><br>Alignment and QC:<br>TrimGalore (version 0.6.5)<br>bwa mem (version 0.7.17)<br>SAMtools (version 0.1.8)<br><br>Calculation of replication timing signal (Log2(E/L)):<br>SAMtools (version 0.1.8)<br>bedtools (version 0.2.26)<br>Repliscan (version 0.1)<br>R (version 3.5.1)<br><br>R packages used in version 3.5.1:<br>preprocessCore (version 1.44.0)

vroom (version 1.5.1)
dplyr (version 1.0.7)
RColorBrewer (version 1.1.3)
gridExtra (version 2.3)
ggpubr (version 0.4.0.999)
ggplot2 (version 3.3.6)
gtable (version 0.3.0)
tidyr (version 1.1.4)
lsa (version 0.73.2)
SnowballC (version 0.7.0)
pheatmap (version 1.0.12)
BSgenome.Hsapiens.UCSC.hg19 (version 1.4.0)
BSgenome (version 1.50.0)
rtracklayer (version 1.42.2)
Biostrings (version 2.50.2)
XVector (version 0.22.0)
GenomicRanges (version 1.34.0)
GenomeInfoDb (version 1.18.2)
IRanges (version 2.16.0)
S4Vectors (version 0.20.1)
BiocGenerics (version 0.28.0)
org.Hs.eg.db (version 3.7.0)
TxDb.Hsapiens.UCSC.hg19.knownGene (version 3.2.2)
GenomicFeatures (version 1.34.8)
AnnotationDbi (version 1.44.0)
Biobase (version 2.42.0)
BSgenome.Hsapiens.UCSC.hg38 (version 1.4.1)
TxDb.Hsapiens.UCSC.hg38.knownGene (version 3.4.0)
circlize (version 0.4.13)
ComplexHeatmap (version 2.5.4)
dendextend (version 1.15.1)
ggdendro (version 0.1.23)
cowplot (version 1.1.1)
ggridges (version 0.5.3)
hdp (version 0.1.5)
deconstructSigs (version 1.9.0)

R packages used in version 4.0.2:
rjson (version 0.2.20)
EnhancedVolcano (version 1.6.0)
edgeR (version 3.30.3)
limma (version 3.44.3)
DESeq2 (version 1.28.1)
matrixStats (version 0.60.0)
optparse (version 1.6.6)
tximport (version 1.16.1)
forcats (version 0.5.1)
stringr (version 1.4.0)
dplyr (version 1.0.7)
purrr (version 0.3.4)
readr (version 2.0.0)
tidyr (version 1.1.3)
tidyverse (version 1.3.1)
data.table (version 1.14.0)
tibble (version 3.1.3)
fst (version 0.9.4)
lsa (version 0.73.2)
vroom (version 1.5.3)
pheatmap (version 1.0.12)
ggrepel (version 0.9.1)
ggbeeswarm (version 0.6.0)
beeswarm (version 0.4.0)
ggpubr (version 0.4.0)
gtable (version 0.3.0)
gridExtra (version 2.3)
ggplot2 (version 3.3.5)
gplots (version 3.1.1)
ComplexHeatmap (version 2.4.3)

RColorBrewer (version 1.1-2)
writexl (version 1.4.0)
readxl (version 1.3.1)
topGO (version 2.40.0)
graph (version 1.66.0)
Homo.sapiens (version 1.3.1)
GO.db (version 3.11.4)
OrganismDbi (version 1.30.0)
biomaRt (version 2.44.4)
GSVA (version 1.36.3)
org.Hs.eg.db (version 3.11.4)
TxDb.Hsapiens.UCSC.hg19.knownGene (version 3.2.2)
GenomicFeatures (version 1.40.1)
AnnotationDbi (version 1.50.3)
Biobase (version 2.48.0)
BSgenome (version 1.56.0)
rtracklayer (version 1.48.0)
Biostrings (version 2.56.0)
XVector (version 0.28.0)
GenomicRanges (version 1.40.0)
GenomeInfoDb (version 1.24.2)
IRanges (version 2.22.2)
S4Vectors (version 0.26.1)
BiocGenerics (version 0.34.0)
ggbreak (version 0.0.9)

python packages used in version 3.9.5
pandas (version 1.2.4)
matplotlib (version 3.3.4)
seaborn (version 0.11.1)
numpy (version 1.19.4)
pyreadr (version 0.4.2)

All code to reproduce the figures is made available under https://zenodo.org/doi/10.5281/zenodo.10950672

For manuscripts utilizing custom algorithms or software that are central to the research but not yet described in published literature, software must be made available to editors and reviewers. We strongly encourage code deposition in a community repository (e.g. GitHub). See the Nature Portfolio guidelines for submitting code & software for further information.

Data

Policy information about availability of data

All manuscripts must include a data availability statement. This statement should provide the following information, where applicable:

- Accession codes, unique identifiers, or web links for publicly available datasets
- A description of any restrictions on data availability
- For clinical datasets or third party data, please ensure that the statement adheres to our policy

Processed data to reproduce the analyses of this study including the replication timing signal data in 50 kb bins for the 31 cell lines analysed in this study can be accessed via https://zenodo.org/doi/10.5281/zenodo.10950672. This repository does not include data from the Genomics England lung cohort due to restricted access. The Genomics England lung cohort is part of the 100,000 Genomes Project whose data are held in a secure research environment and are only available to registered users. For further information on how to obtain access visit https://www.genomicsengland.co.uk/research/academic. Somatic variants for the 560 WGS breast cancer dataset are available on the International Cancer Genome Consortium Data Portal (https://dcc.icgc.org/) and were retrieved via ftp://ftp.sanger.ac.uk/pub/cancer/Nik-ZainalEtAl-560BreastGenomes/. Supplementary files from Nik-Zainal et al. 2 were downloaded for additional information, including clinical data. The TCGA data were retrieved through database of Genotypes and Phenotypes (dbGaP) authorisation (accession no. phs000854.v3.p8). Information about TCGA and the investigators and institutions who constitute the TCGA research network can be found at https://cancergenome.nih.gov/. The accession numbers for the raw Repli-seq data of the 16 cell lines downloaded from ENCODE are listed in Supplementary Table 2. The accession numbers for the Hi-C data downloaded from ENCODE are provided in Supplementary Table 3. The raw data of the 13 in-house repli-sequenced cell lines has been made publicly available on SRA under the BioProject accession number PRJNA1096133. The raw data of the 13 in-house repli-sequenced cell lines has been made publicly available on SRA under the BioProject accession number PRJNA1096133. The raw data of the TRACERx PDCs (from the TRACERx study) used during this study has been deposited at the European Genome–phenome Archive (EGA), which is hosted by The European Bioinformatics Institute (EBI) and the Centre for Genomic Regulation (CRG) under the accession code EGAS00001007773 and is under controlled access due to its nature and commercial licences. Specifically, data is available through the Cancer Research UK & University College London Cancer Trials Centre (ctc.tracerx@ucl.ac.uk) for academic non-commercial research purposes only and is subject to review of a project proposal by the TRACERx data access committee, entering into an appropriate data access agreement and subject to any applicable ethical approvals. A response to the request for access is typically provided within 10 working days after the committee has received the relevant project proposal and all other required information. The access to the data will expire on the third anniversary of the effective date of the agreement.

Research involving human participants, their data, or biological material

Policy information about studies with human participants or human data. See also policy information about sex, gender (identity/presentation), and sexual orientation and race, ethnicity and racism.

Reporting on sex and gender	In our analysis we have not distinguished between sex or gender, which is why we are not mentioning these terms in the title, abstract or other sections of our manuscript. The patient data analysed in this study were downloaded from public sources. Information about sex and gender of these patients can be found in the corresponding publications and data portals.
Reporting on race, ethnicity, or other socially relevant groupings	In our analysis we have not distinguished between race, ethnicity or other socially relevant groupings, which is why we are not mentioning these terms in the title, abstract or other sections of our manuscript. The patient data analysed in this study were downloaded from public sources. Information about race, ethnicity and other social groupings of these patients can be found in the corresponding publications and data portals.
Population characteristics	In our study, we mainly analysed replication timing sequencing data from lung and breast cancer cell lines, whole-genome sequencing data of 952 cancer genomes derived from the 100,000 Genomes Project (Caulfield, M. et al. The National Genomics Research and Healthcare Knowledgebase. 2019. doi:10.6084/M9.FIGSHARE.4530893.V5) and Nik-Zainal et al. (Nik-Zainal, S. et al. Landscape of somatic mutations in 560 breast cancer whole-genome sequences. Nature 534, 47–54. 2016). and bulk RNA sequencing data of lung and breast tumours from TCGA. The genomic and transcriptomic data from tumours analysed in this study were derived from public repositories. Further information about the selection of patients within these studies can be found in the corresponding publications and data portals.<br><br>Paired WGS and replication timing sequencing data of two patient derived cell lines (PDCs) from patients enrolled in the TRACERx study were analysed in scope of this publication. These two PDCs were the only PDCs established in our laboratory at the time point of study design. The PDCs were derived from patients with the following characteristics:<br>* CRUK0557: 64-year-old, Male, White- British<br>* CRUK0977: 77-year-old, Female, White- British<br>Further information about the TRACERx study can be found in the following publication https://doi.org/10.1038/s41586-023-05783-5
Recruitment	No recruitment has been performed in scope of this study. The patient data analysed in this study were downloaded from public resources.
Ethics oversight	This study complies with all relevant ethical regulations required by the University College London Cancer Institute and the Francis Crick Institute.

Note that full information on the approval of the study protocol must also be provided in the manuscript.

Field-specific reporting

Please select the one below that is the best fit for your research. If you are not sure, read the appropriate sections before making your selection.

- [x] Life sciences
- [ ] Behavioural & social sciences
- [ ] Ecological, evolutionary & environmental sciences

For a reference copy of the document with all sections, see nature.com/documents/nr-reporting-summary-flat.pdf

Life sciences study design

All studies must disclose on these points even when the disclosure is negative.

Sample size	This study is based on a collection of different publicly available datasets and some in-house replication timing sequencing data from cell lines. No overall sample size calculations were performed for this study. Instead as much replication timing sequencing data as possible and large amounts of WGS data that fulfilled the study requirements were used.<br><br>All replication timing sequencing data available on ENCODE at the time of study design were downloaded. This dataset included some LUAD and BRCA cell lines but no cell line derived from the tissue of origin of LUAD and BRCA tumours. Therefore, to be able to investigate alterations in the replication timing profile between non-malignant and cancerous cells we performed replication timing sequencing for one cell line derived from the tissue of origin of LUAD tumours (T2P) and one from the reported tissue of origin of BRCA tumours (HMEC). To account for intra tumour heterogeneity we performed replication timing sequencing for further LUAD and BRCA cell lines which resulted in the classification of altered replication timing regions in 4 LUAD and 4 BRCA cell lines relative to their normal reference. Additionally, two non malignant lung cell lines and one breast cell line, that were not derived from the tissue of origin, were included in the analysis to explore the effect of differences in the replication timing between tissues and cell differentiation states on the identification of altered replication timing in LUAD and BRCA.<br><br>For only 4 of the analysed LUAD and BRCA cell lines, including their normal references, Hi-C data was publicly available on ENCODE.<br><br>To explore the relationship between the genomic and transcriptomic landscape and alterations in the replication timing profile, all 470 LUAD tumours whose data was available in the Genomics England research environment and that passed our QC checks at the time of study design were analysed. All 482 lobular and ductal BRCA tumours from the publication by Nik-Zainal et al. in Nature 2016 (doi:10.1038/nature17676) were included. Transcriptomic data of 1347 LUAD and BRCA tumours from TCGA of which 149 also provided data from adjacent normal tissue

	were analysed. Copy number data of 1474 LUAD and BRCA tumours from TCGA were analysed in this study.<br><br>To apply a paired analysis of WGS and replication timing sequencing from the same sample, two PDCs derived from patients from the TRACERx study were used. Those were the only two PDCs that were established in our lab at the time of study design for which such an analysis was possible.
Data exclusions	Samples which failed quality control metrics were excluded from the analysis.
Replication	Biological replicates of three cell lines were performed to validate the consistency of our Repli-seq protocol and bioinformatic pipeline, and all attempts at replication were successful.
Randomization	No randomization was conducted in this study, because when samples were grouped together, the grouping strategy was based on the pathological subtype of each tumour or cell line, rather than randomization.
Blinding	No blinding was conducted in this study, because no treatment or other interference were involved in our analyses.

Reporting for specific materials, systems and methods

We require information from authors about some types of materials, experimental systems and methods used in many studies. Here, indicate whether each material, system or method listed is relevant to your study. If you are not sure if a list item applies to your research, read the appropriate section before selecting a response.

Materials & experimental systems

n/a	Involved in the study	
☐	☒	Antibodies
☐	☒	Eukaryotic cell lines
☒	☐	Palaeontology and archaeology
☒	☐	Animals and other organisms
☒	☐	Clinical data
☒	☐	Dual use research of concern
☒	☐	Plants

Methods

n/a	Involved in the study	
☒	☐	ChIP-seq
☐	☒	Flow cytometry
☒	☐	MRI-based neuroimaging

Antibodies

Antibodies used	Mouse anti-BrdU antibody (Monoclonal, Clone B44. BD Biosciences; 347580): 12.5µg/ml at stock; 40µl mouse anti-BrdU antibody at a concentration of 12.5µg/ml was used for each sample at 560 µl to achieve a final concentration of 0.83 µg/ml;<br>Goat anti-mouse secondary antibody (IgG-Alexa Fluor 488. Abcam; ab150129): 1mg/ml at stock; 20µg (i.e. 20µl) goat anti-mouse secondary antibody at a concentration of 1mg/ml was used for each sample to achieve a final concentration of 0.03 µg/µl.
Validation	These two antibodies were bought from two companies. The relevant validation information can be found on the website of each company:<br>Mouse anti-BrdU antibody (Monoclonal, Clone B44. BD Biosciences; 347580): https://www.bdbiosciences.com/content/bdb/paths/generate-tds-document.us.347580.pdf<br>Goat anti-mouse secondary antibody (IgG-Alexa Fluor 488. Abcam; ab150129): https://www.abcam.com/products/secondary-antibodies/goat-mouse-igg-hl-alexa-fluor-488-ab150113.html

Eukaryotic cell lines

Policy information about [cell lines and Sex and Gender in Research]

Cell line source(s)	The pulmonary alveolar epithelial type II cells (T2P), the human mammary epithelial cells (HMEC, known as hTERT-HME 1), six lung cancer cell lines (A549, H1650, H1792, H2009, H520 and H2170) as well as MCF10A, SK-BR3 and MDA453 were provided by Cell Services at the Francis Crick Institute in London. SW900 was purchased from ATCC (https://www.atcc.org/). The TT1 (pulmonary alveolar epithelial type I cells) cell line was courtesy of Dr Michele Chiappi and Professor Terry Tetley (National Heart and Lung Institute, Imperial College London, UK), the information of which has been published here (Kemp, S. J. et al. Immortalization of human alveolar epithelial cells to investigate nanoparticle uptake. Am. J. Respir. Cell Mol. Biol. 39, 591–597, 2008). The two PDCs from TRACERx patients were initiated in our lab.
Authentication	For Cell Authentication we use STR (Short Tandem Repeat) Profiling for all our Human cell lines using the Promega PowerPlex16HS system. This profile is compared back to any available on commercial cell banks (such as ATCC). We confirm the species is correct using a primer system based on the Cytochrome C Oxidase Subunit 1 gene from mitochondria – we call this test Species ID. Authentication is carried out in house within the Francis Crick Institute.
Mycoplasma contamination	For Mycoplasma screening we primarily use two different tests – Agar Culture (which involves culturing any mycoplasma that may be present in the cell culture on specialised agar) and Fluorescent staining using the Hoescht Stain. A third detection method, the PCR mycoplasma test (ATCC), is used on occasion when a rapid result is required. Mycoplasma test is carried out

	in house routinely within the Francis Crick Institute. We confirm that all cell lines in our study were tested negative for mycoplasma contamination.
Commonly misidentified lines (See ICLAC register)	No commonly misidentified cell lines were included in this study.

Plants

Seed stocks	No plants were involved in our study.
Novel plant genotypes	No plants were involved in our study.
Authentication	No plants were involved in our study.

Flow Cytometry

Plots

Confirm that:

- [x] The axis labels state the marker and fluorochrome used (e.g. CD4-FITC).
- [x] The axis scales are clearly visible. Include numbers along axes only for bottom left plot of group (a 'group' is an analysis of identical markers).
- [x] All plots are contour plots with outliers or pseudocolor plots.
- [x] A numerical value for number of cells or percentage (with statistics) is provided.

Methodology

Sample preparation	Asynchronized cells grew in flasks for at least 48h to achieve at least 107 cells with a confluency of less than 80% and were labelled with bromodeoxyuridine 5-bromo-2'-deoxyuridine (BrdU) for 2 hours at 37 ºC in a CO2 incubator in the dark. Cells were then harvested and washed using ice-cold PBS twice each time after spinning down (speed, 340xg for 5 min at 4 ºC in the dark). Cell pellets were fixed in a mixture of 7.5ml ice-cold 100% ethanol and 2.5ml PBS containing 2% (v/v) FBS. The fixed cell pellets were incubated on ice for at least 30 min or stored at -20 ºC.<br><br>After washing twice using ice-cold washing buffer (PBS with 1% FBS), DNA contents in the fixed cell pellets were washed twice using ice-cold washing buffer (PBS with 1% FBS) and stained using 200 µl propidium iodide (PI) buffer per million cells which contained 50µl 50µg/ml ribonuclease A (Sigma; P4170) and 150µl 100µg/ml PI (Sigma; R5125). Prior to sorting cells using fluorescence-activated cell sorting (FACS), to get a proper single cell suspension, the cell pellets were disaggregated by passing through a 25G needle using a 1ml syringe and filtered through a 40um nylon mesh to remove any clumps or aggregates.
Instrument	BD FACS Aria II
Software	No other softwares were involved. All sorted cells were applied to further DNA extraction and other steps in Repli-seq protocol. Repli-seq data were run and analysed on Rstudio as aforementioned.
Cell population abundance	The aim of FACS in our study was to select the asynchronized cell lines which were in early or late S phase at the time of fixation. Then the sorted samples were rerun through the instrument one by one to test the range of their DNA contents (PI components). When the DNA contents in early verusus late replicating cells were consistent with what we sorted, the purity of sorted cells was considered as passing quality control.
Gating strategy	Cells were initially gated using forward scatter (FSC) versus side scatter (SSC) to exclude debris and aggregates. Subsequently, sequential gating strategies were employed to identify single cells, including gating based on FSC-A versus FSC-H, SSC-A versus SSC-W, and propidium iodide (PI)-A versus PI-W to exclude apoptotic cells and doublets further. The G0/G1 and G2/M phases were distinguished by the peak of PI staining intensity, incorporating DNA content with cell size (FSC-A) as a reference. To ensure accuracy, early-S and late-S phases were equally gated between the G0/G1 and G2/M phases, while the edges of each population were avoided to minimise potential artefacts.

- [x] Tick this box to confirm that a figure exemplifying the gating strategy is provided in the Supplementary Information.
